# Single-Molecule-Based, Label-Free Monitoring of Molecular Glue Efficacies for Promoting Protein–Protein Interactions Using YaxAB Nanopores

**DOI:** 10.1021/acsnano.4c11436

**Published:** 2024-11-01

**Authors:** Minju Ryu, Sohee Oh, Ki-Baek Jeong, Sungbo Hwang, Jin-Sik Kim, Minji Chung, Seung-Wook Chi

**Affiliations:** † Disease Target Structure Research Center, Division of Biomedical Research, Korea Research Institute of Bioscience and Biotechnology (KRIBB), Daejeon 34141, Republic of Korea; ‡ Department of Proteome Structural Biology, KRIBB School of Bioscience, University of Science and Technology, Daejeon 34113, Republic of Korea; § Critical Diseases Diagnostics Convergence Research Center, KRIBB, Daejeon 34141, Republic of Korea; ∥ School of Pharmacy, Sungkyunkwan University, Suwon, Gyeonggi 16419, Republic of Korea

**Keywords:** Biological nanopore, molecular glue, protein−protein interaction, single-molecule, label-free detection

## Abstract

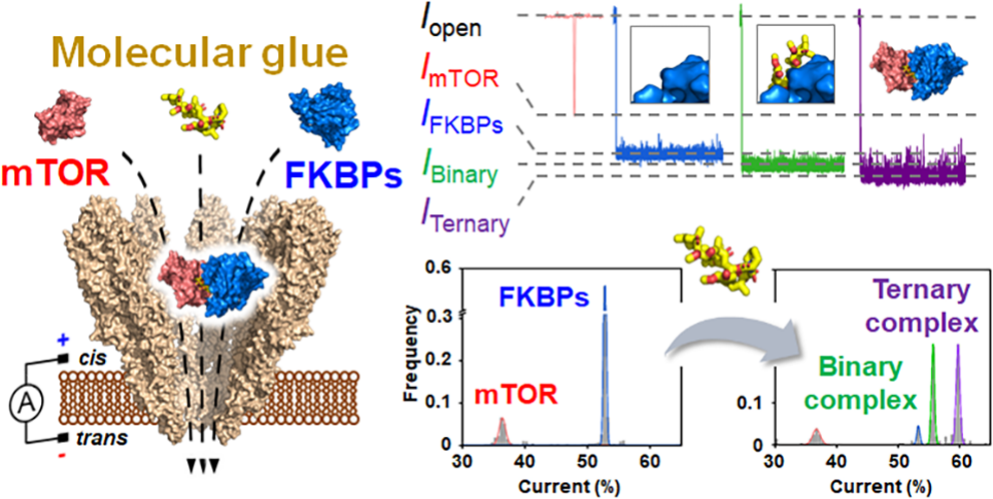

Modulating protein–protein interactions (PPIs) is an attractive strategy in drug discovery. Molecular glues, bifunctional small-molecule drugs that promote PPIs, offer an approach to targeting traditionally undruggable targets. However, the efficient discovery of molecular glues has been hampered by the current limitations of conventional ensemble-averaging-based methods. In this study, we present a YaxAB nanopore for probing the efficacy of molecular glues in inducing PPIs. Using YaxAB nanopores, we demonstrate single-molecule-based, label-free monitoring of protein complex formation between mammalian target of rapamycin (mTOR) and FK506-binding proteins (FKBPs) triggered by the molecular glue, rapamycin. Owing to its wide entrance and adjustable pore size, in combination with potent electro-osmotic flow (EOF), a single funnel-shaped YaxAB nanopore enables the simultaneous detection and single-molecule-level quantification of multiprotein states, including single proteins, binary complexes, and ternary complexes induced by rapamycin. Notably, YaxAB nanopores could sensitively discriminate between the binary complexes or ternary complexes induced by rapamycin and its analogues, despite the subtle size differences of ∼122 or ∼116 Da, respectively. Taken together, our results provide proof-of-concept for single-molecule-based, label-free, and ultrasensitive screening and structure–activity relationship (SAR) analysis of molecular glues, which will contribute to low-cost, highly efficient discovery, and rational design of bifunctional modality of drugs, such as molecular glues.

Protein–protein interactions (PPIs) are crucial in regulating various biological processes.^1^ Modulating PPIs is regarded as a promising strategy in drug development. Although drug development has been intensively focused on PPI inhibitors, targeting most PPIs within the human interactome remains challenging^2^ because the protein binding sites involved in these interactions often feature flat or protruding surfaces. Molecular glues are bifunctional small-molecule drugs, which offer an approach for drug development by creating or stabilizing PPIs through the formation of ternary complexes.^3^ When a molecular glue binds to its target protein, the resulting binary complexes create a temporarily specific surface or pocket on the distorted surface^4^ that facilitates the binding of a guest protein, ultimately forming a ternary complex. Unlike traditional PPI inhibitors, molecular glues may not require strong binding affinity, as they do not compete directly with native guest proteins.^5^ Thus, molecular glue-induced PPI modulation represents an innovative strategy for targeting traditionally undruggable proteins in drug development.^6^

Recently, there has been increasing attention on molecular glue-based PPI modulation, highlighted by several successful clinical cases including IMiDs, thalidomide (Thalomid), lenalidomide (Revlimid), and pomalidomide (Pomalyst). However, the discovery of molecular glues poses challenges, as most of them have been identified through serendipitous discoveries.^7,8^ Cell-based assays such as time-resolved Forster resonance energy transfer (TR-FRET)^9^ and nanoluciferase bioluminescence resonance energy transfer (NanoBRET),^10^ as well as in vitro methods including nuclear magnetic resonance (NMR)^11^ and surface plasmon resonance (SPR),^12^ are used for screening of molecular glues. Despite advancements in these techniques, the current screening methods of molecular glues suffer from several shortcomings,^13^ including inaccurate evaluations of drug efficacies or unwanted side effects due to protein labeling or tagging.^14^ Therefore, it is necessary to develop highly efficient approaches for screening molecular glues with label-free, accurate, ultrasensitive, and high-throughput measurements.

Nanopore technology utilizes nanometer-sized pores that allow ion flow under an applied voltage bias. By measuring current changes at the pico-ampere level as an analyte passes through the pore or is trapped inside, various characteristics of a single-molecule analyte can be elucidated.^15^ Nanopore sensing has primarily relied on the principle of electrophoresis, which drives charged biomolecules into the nanopore. Recently, nanopore sensing with electro-osmotic flow (EOF) has been developed, enabling control of water molecule movement within the pores. In biological nanopores such as MspA,^16^ FraC,^17^ ClyA,^18^ PlyAB,^19^ CytK,^20^ and YaxAB,^21,22^ EOF-based sensing enables sensitive observation of single-molecule proteins, regardless of their net charges. Our previous study showed that YaxAB nanopores, with their funnel-shaped wide entrance and adjustable pore size, could detect a broad range of target proteins.^21^ Furthermore, the high density of ions within the narrow constriction of a YaxAB nanopore enabled highly sensitive detection of tiny ionic flow changes in response to subtle variations in target proteins. This capability facilitated single-molecule analysis of folded proteins, PPIs and protein-drug interactions (PDIs).

Rapamycin, an FDA-approved immunosuppressant for organ transplant patients,^23,24^ is a molecular glue that facilitates the interaction between the mammalian target of rapamycin (mTOR)-FKBP12-rapamycin-binding (FRB) domain and FK506-binding proteins (FKBPs). mTOR is a serine-threonine kinase involved in regulating cell proliferation, apoptosis, metabolism, and autophagy.^25^ FKBPs, including FKBP12 and FKBP25, are a large family of proteins that mediate the immunosuppressive behavior of FK506.^26^ Given these roles, the interaction between the mTOR-FRB domain and FKBPs has garnered significant attention as a PPI target for immunosuppression and cancer therapy.^27^ In this study, we developed a single-molecule-based, label-free approach for analyzing the efficacy of molecular glues in promoting protein–protein interactions by using a biological nanopore. Utilizing YaxAB nanopores, we demonstrated single-molecule, label-free monitoring of interactions between the mTOR-FRB domain (hereafter referred to as mTOR) and FKBPs triggered by molecular glues, rapamycin, and its analogs. Notably, a single YaxAB nanopore could simultaneously detect and sensitively discriminate among molecular glue-induced ternary complexes, binary complexes, and individual proteins (mTOR and FKBPs). Taken together, our results suggest that YaxAB nanopores represent a robust platform for single-molecule-based, label-free, and ultrasensitive screening, enabling the highly efficient discovery of molecular glues.

## Results and Discussion

### Characterization of YaxAB-C_9_ Nanopores

**Figure 1:**
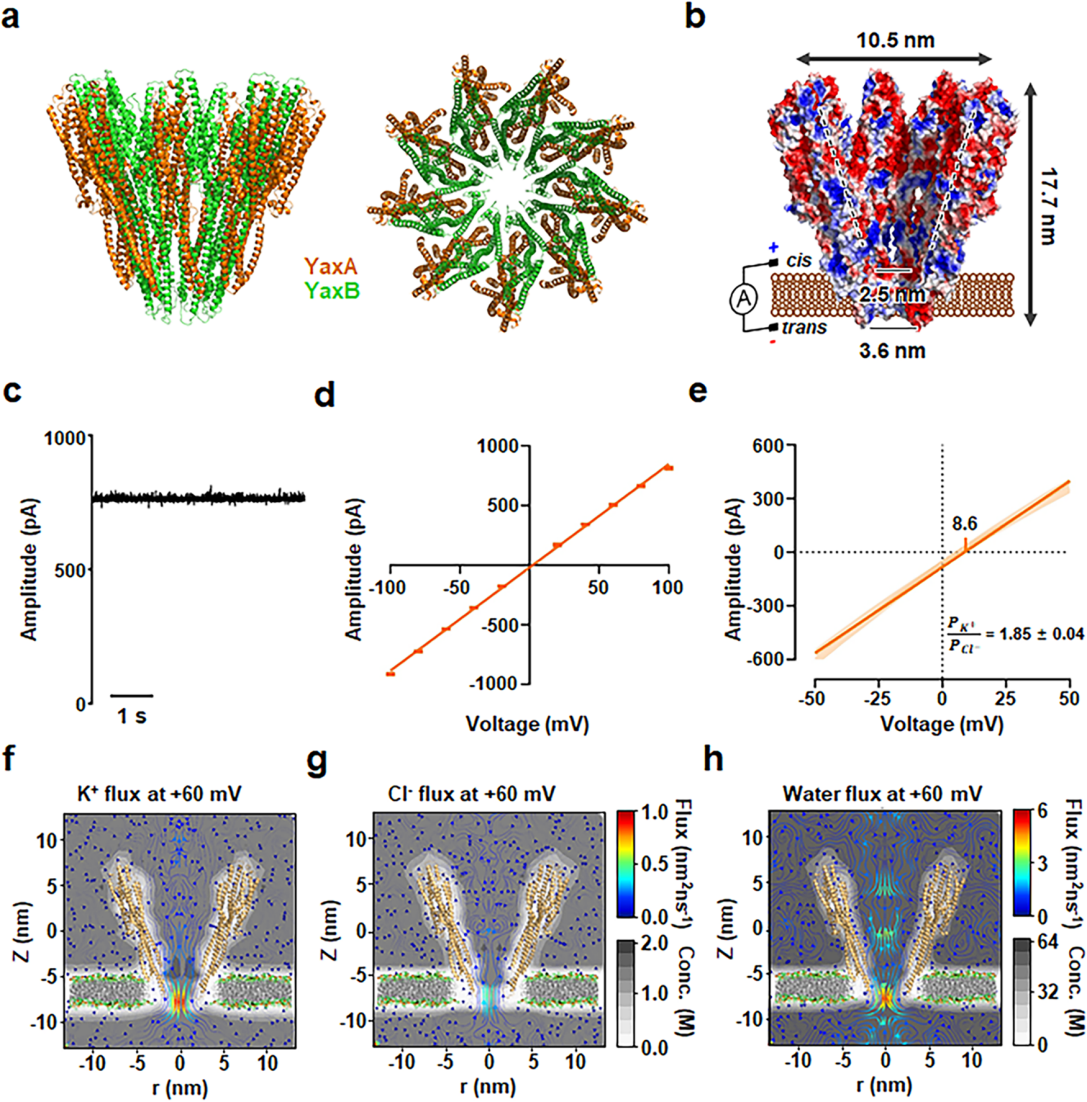
Structure and nanofluidic characteristics of a YaxAB-C_9_ nanopore. (a) Structural model of a YaxAB-C_9_ nanopore, highlighting its views along the C_9_ symmetry axis and side views. YaxA and YaxB are colored orange and green, respectively. (b) Cross-section of a YaxAB-C_9_ nanopore. The electrostatic potential of the surface is computed by the adaptive Poisson–Boltzmann solver. The funnel-shaped geometry of the inner surface is shown as black dotted lines. (c) Single-pore insertion of a YaxAB-C_9_ nanopore. The current trace was filtered with a Bessel (8-pole) filter at 1 kHz. (d) Current–voltage (*I*–*V*) plot of a YaxAB-C_9_ nanopore. Data are presented as mean ± SD, *n* = 3 independent replicates for YaxAB-C_9_ nanopores. (e) Reversal potential, showing cation selectivity for a YaxAB-C_9_ nanopore. The reversal potential value is 8.6 ± 0.3 mV. All reversal potentials were measured under asymmetric salt conditions (2 and 0.5 M KCl on the *trans* and *cis* sides, respectively) in a pH 8.0 buffer. Data are presented as mean ± SD, *n* = 3 independent replicates for each YaxAB-C_9_ nanopore. (f–h) MD simulation of a YaxAB-C_9_ nanopore at +60 mV voltage bias and in 1 M KCl. The YaxAB nanopore exhibits a comparably strong directional K^+^ and water flow from the *cis* to the *trans* side against Cl^–^ flow.

In a previous study,^21^ YaxAB nanopores of three different sizes were separated using native gel electrophoresis and gel extraction. These nanopores are termed YaxAB-C_8_ (16-mer), YaxAB-C_9_ (18-mer), and YaxAB-C_10_ (20-mer), based on the number of heterodimers (C*_n_*; YaxA + YaxB) within the oligomer (Figure 1a). In this study, we employed the YaxAB-C_9_ nanopore, which is composed of nine heterodimers with apparent C_9_ symmetry, to analyze a broader range of folded protein sizes compared to the YaxAB-C_8_ nanopore.^21^ Using structural modeling as described previously,^28^ we generated the structure of the YaxAB-C_9_ nanopore, which features an ∼18 nm long “coffee-dripper”-shaped lumen, with a *cis* entry of 10.5 nm, a *trans* entry of 3.6 nm, and a constriction diameter of 2.5 nm (Figure 1b). The YaxAB-C_9_ pore was inserted into artificial lipid membranes and exhibited a stable open conductance of 8.1 ± 0.1 nS under constant conditions of 1 M KCl and pH 8.0 (Figure 1c). This pore follows Ohm’s law (Figure 1d), indicating stability within the lipid membrane. The ohmic behavior of the YaxAB-C_9_ pore was confirmed by measuring current flow across a voltage range of −100 to +100 mV. To analyze the ion selectivity of the YaxAB-C_9_ pore, we applied an asymmetric salt concentration (*trans* 2 M KCl, *cis* 0.5 M KCl) and pH 8.0. The reversal potential corresponding to the current shift was measured, and the ion selectivity of the YaxAB-C_9_ pore was calculated using the Goldman–Hodgkin–Katz equation (Figure 1e).^29^ The reversal potential of the YaxAB-C_9_ pore was 8.6 ± 0.3 mV, demonstrating strong cation selectivity with a *P*_K^+^_/*P*_Cl^–^_ value of 1.85 ± 0.04. Based on previous results of YaxAB-C_8_ pore,^21^ the cation selectivity of the YaxAB-C_9_ pore suggests that K^+^ ions and water molecules move from the *cis* to *trans* side of the pore.

To characterize the nanofluidic properties of the YaxAB-C_9_ pore, molecular dynamics (MD) simulations were conducted to analyze the flow of K^+^ ions, Cl^–^ ions, and water molecules through the pore. The simulations provided local distribution profiles for ions and water within the YaxAB-C_9_ pore under an applied voltage of +60 mV (Figure 1f–h). At this voltage bias, the flux of K^+^ ions (0.86 nm^2^·ns^–1^) and water molecules (4.96 nm^2^·ns^–1^) was highly concentrated in the constriction region of the YaxAB-C_9_ pore, whereas the flux of Cl^–^ ions (0.45 nm^2^·ns^–1^) was significantly lower than those of K^+^ ions and waters. These findings confirm the strong cation selectivity of the YaxAB-C_9_ pore and the presence of a potent EOF through the pore from the *cis* to *trans* side, consistent with the results from the electrical measurements. The accumulation of cations in the narrow constriction of the funnel-shaped YaxAB-C_9_ nanopore may reflect the attraction of K^+^ ions to the negatively charged constriction region.

### Single-Molecule Detection of Molecular Glue-Induced PPI Using YaxAB Nanopores

**Figure 2:**
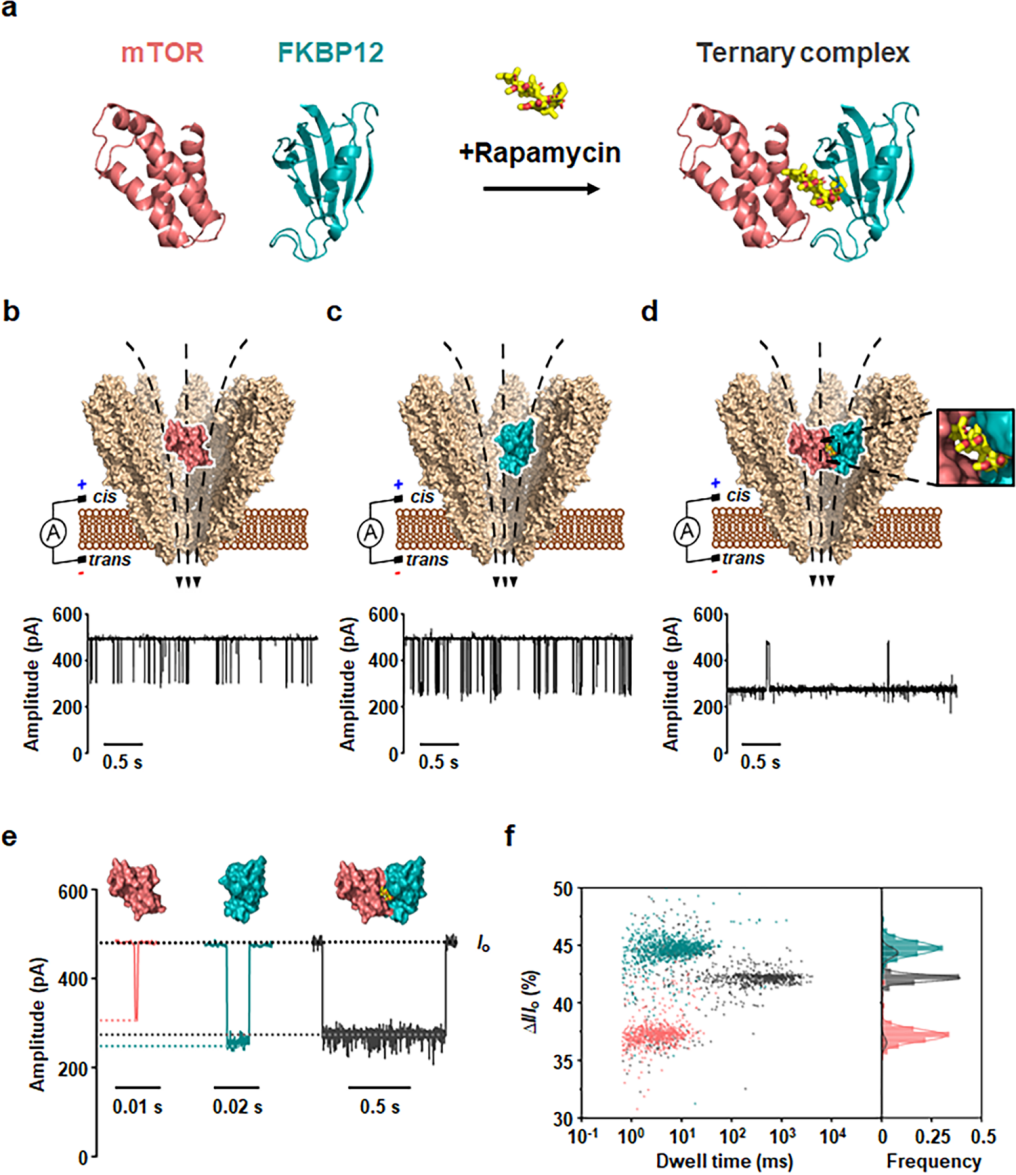
Detection of rapamycin-induced mTOR-FKBP12 interaction using YaxAB nanopores. (a) Formation of the rapamycin-induced mTOR-FKBP12-rapamycin ternary complex. (b–d) Schematic illustrations and representative current traces corresponding to the detection of mTOR (b), FKBP12 (c), and mTOR-FKBP12-rapamycin (1:1:10) ternary complex (d) using YaxAB nanopores. (e) Representative event signals consisting of blockade current and dwell time corresponding to mTOR, FKBP12, and mTOR-FKBP12-rapamycin ternary complex. (f) Overlaid 2D scatter plots (Δ*I*/*I*_o_-versus-dwell time) for the detection of mTOR (salmon), FKBP12 (cyan), and mTOR-FKBP12-rapamycin (1:1:10) ternary complex (black), respectively. All electrical measurements were performed at an applied potential of +60 mV. Current traces were filtered with a Bessel (8-pole) filter at 1 kHz.

The therapeutic efficacy of molecular glues is mediated by PPIs induced with them. To monitor the efficacy of molecular glues, it is crucial to detect the molecular-glue-mediated formation of ternary complexes from individual proteins. In this study, we employed rapamycin, a well-known molecular glue, along with its target proteins, mTOR and 12 kDa FK506-binding protein (FKBP12). Prior to nanopore experiments, we confirmed the formation of a ternary complex among mTOR, FKBP12, and rapamycin by using NMR spectroscopy. Specifically, we performed two-dimensional (2D) ^1^H–^15^N heteronuclear single quantum correlation (HSQC) NMR experiments with ^15^N-labeled mTOR in the absence and presence of FKPB12 and rapamycin (Figure S1). Initially, no change in the chemical shift was observed in the ^1^H–^15^N cross-peaks of mTOR upon the addition of FKBP12 alone, indicating no interaction between them without rapamycin. However, upon the addition of rapamycin, significant changes in the ^1^H–^15^N cross-peaks of mTOR were observed, confirming that rapamycin binds to mTOR. Furthermore, when both FKBP12 and rapamycin were added, most of the ^1^H–^15^N cross-peaks of mTOR disappeared due to severe line broadening of the NMR resonances. These observed chemical shift perturbations demonstrated a direct interaction between mTOR and FKBP12, indicating that rapamycin induces their interaction. This finding is consistent with the protein complex structure previously determined by X-ray crystallography (Figure 2a).^30^

Next, we performed nanopore measurements using YaxAB-C_9_ nanopores to analyze individual target proteins (mTOR or FKBP12) and the ternary complex (mTOR-FKBP12-rapamycin). Although these three protein analytes differ in size and net charge (Figure S2) (mTOR, 12.3 kDa, −1.7e; FKBP12, 13.0 kDa, −0.6e; mTOR-FKBP12-rapamycin, 26.2 kDa, −1.8e), all were successfully trapped inside the YaxAB-C_9_ nanopore via EOF-driven flow, regardless of their size and net charges (Figure 2b–d). Since proteins can either be captured within the nanopore or translocate through the nanopore in a voltage-dependent manner,^17^ we determined the threshold voltage required for the passage of each protein by applying voltages ranging from +60 to +140 mV. Negatively charged mTOR exhibited nanopore translocations at voltages above +100 mV, while nearly neutral FKBP12 translocated at voltages above +80 mV. The observed voltage-dependent decrease of dwell times (Figures S3 and S4) supported their translocation across YaxAB-C_9_ nanopore. It is notable that mTOR and FKBP12 with a larger size than the constriction could translocate across the pore, which might arise from deformation or structural flexibility of transmembrane α-helices in the YaxAB-C_9_ nanopore during the translocation. This is consistent with previous observations of translocation of protein analytes with a larger size than the constriction across other α-helical nanopores, ClyA and FraC.^17,31,32^ To achieve optimal trapping of mTOR, FKBP12, and their ternary complex, we selected the +60 mV condition to prevent the proteins from translocating through the nanopore (Figures S3 and S4). This allowed us to avoid the excessively long trapping times of the ternary complex observed at higher voltages and obtain appropriate event frequencies for mTOR, FKBP12, and the ternary complex for statistical analysis. The ionic current blockade (Δ*I*/*I*_o_) and dwell time were used to distinguish the three protein analytes. Under the optimal voltage condition (+60 mV), the current blockade (Δ*I*/*I*_o_) signals of mTOR, FKBP12, and the ternary complex were clearly discriminated (Figure 2e). Additionally, overlaid 2D scatter plots of current blockade (Δ*I*/*I*_o_) versus dwell time revealed distinctly different event populations for the two single proteins and the ternary complex (Figure 2f). The mean Δ*I*/*I*_o_ values for mTOR, FKBP12, and the ternary complex were 37.30 ± 0.34, 44.87 ± 0.65, and 42.18 ± 0.04%, respectively.

In a funnel-shaped structure of YaxAB nanopores with a higher density of ions in the constriction, the current blockade (Δ*I*/*I*_o_) may depend on the position of analytes within the pore, which could be governed by various factors of analytes such as net charge, molecular size, and interaction with the pore. Despite the similar molecular sizes of mTOR (3.2 nm × 4.3 nm × 3.3 nm, 12.3 kDa) and FKBP12 (3.0 nm × 4.6 nm × 3.5 nm, 13.0 kDa), there was a significant difference in mean Δ*I*/*I*_o_ value (37.30 ± 0.34 and 44.87 ± 0.65% for mTOR and FKBP12, respectively). This difference may be attributed to variations in the residence site of the protein analytes within the YaxAB-C_9_ nanopore. For negatively charged analytes undergoing repulsive EPF against the EOF direction, their current blockade could be influenced predominantly by net charge: the negatively charged mTOR (−1.7e at pH 8.0) and mTOR-FKBP12-rapamycin ternary complex (−1.8e at pH 8.0) exhibited a smaller current blockade than nearly neutral FKBP12 (−0.6e at pH 8.0) (Figure 2e). This observation aligns with previous findings of current blockade differences between Bcl-xL and its differentially charged mutants using YaxAB-C_8_ nanopores.^21,31^ Taken together, our results demonstrate that YaxAB-C_9_ nanopores can effectively monitor rapamycin-induced ternary complex formation from individual proteins mTOR and FKBP12.

**Figure 3:**
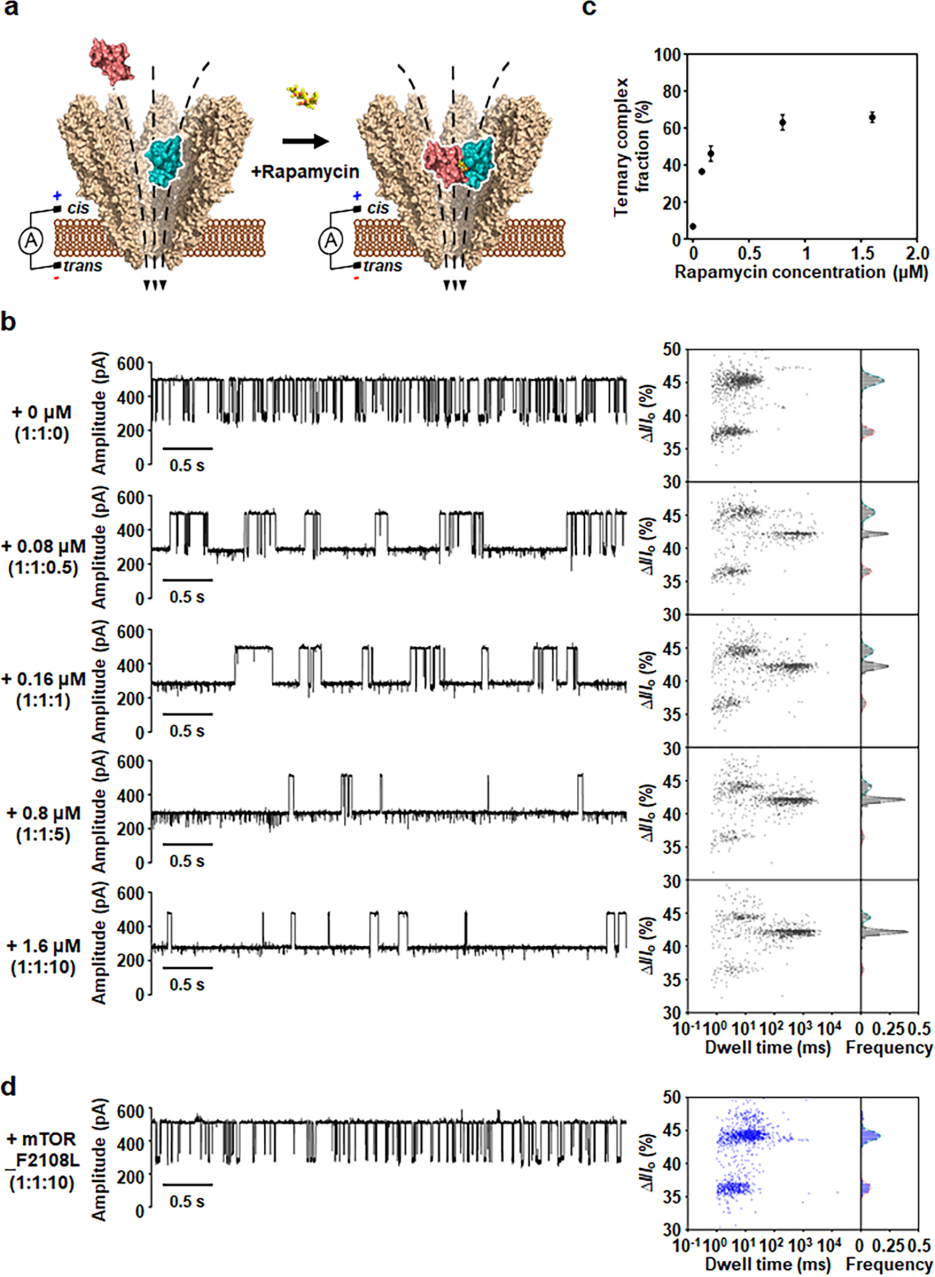
Validation of rapamycin-induced formation of ternary complex. (a) Schematic illustration of the detection of the rapamycin-dependent ternary complex. (b) Current traces and statistical analysis of 2D scatter plot (Δ*I*/*I*_o_ versus dwell time) corresponding to the detection of mTOR and FKBP12 with concentrations of rapamycin from 0 to 1.6 μM. A 2 h preincubation time was given after the addition of rapamycin to prepare each sample of complex. (c) Dose–response curve for the formation of the ternary complex. (d) Current trace and statistical analysis of 2D scatter plot (Δ*I*/*I*_o_ versus dwell time) corresponding to the detection of mTOR_F2108L mutant, FKBP12, and rapamycin at a 1:1:10 molar ratio. The incubated mixture was added to the *cis s*ide of the YaxAB-C_9_ nanopore at various molar ratios. All the electrical measurements were performed at an applied potential of +60 mV. Current traces were filtered with a Bessel (8-pole) filter at 1 kHz.

To confirm that the observed nanopore events of the mixture (black, Figure 2e,f) are specifically caused by rapamycin-induced ternary complexes, we conducted further nanopore experiments at varying rapamycin concentrations (Figure 3a,b). In the absence of rapamycin, the nanopore events from ternary complexes were not observed, except for those from individual proteins. The concentration ratio used in the experiment was fixed at the 1:1 molar ratio for mTOR and FKBP12 (160 nM), while rapamycin concentrations were 0, 0.08, 0.16, 0.8, and 1.6 μM. As a result, event populations of the ternary complex increased gradually and those of individual proteins decreased with increasing rapamycin concentrations (Figure 3b). These data generated a dose–response curve for rapamycin (Figure 3c), which could be useful for hit validation and mechanism-of-action analysis. Furthermore, nanopore events were measured with a rapamycin-binding defective mTOR mutant (mTOR_F2108L)^33,34^ to disrupt rapamycin binding. Unlike wild-type mTOR, the nanopore events from rapamycin-induced ternary complex were not observed in the 2D scatter plot of mTOR_F2108L (Figure 3d), indicating that the formation of a ternary complex arises specifically from rapamycin binding. Taken together, our results showed that YaxAB-C_9_ nanopores can be used to quantitatively monitor the formation of molecular glue-induced ternary complexes from individual proteins at a single-molecule level.

### Single-Molecule Detection of Protein–Molecular Glue Interactions Using YaxAB Nanopores

Monitoring direct physical interactions between drugs and their cognate targets is essential in drug discovery processes. The binding of a molecular glue to its target protein is the initial step in the formation of ternary complexes. Building on our previous protein–drug interaction (PDI) analysis using YaxAB-C_8_ nanopores,^21^ we investigated protein–molecular glue interactions at the single-molecule level using YaxAB-C_9_ nanopores. At an applied voltage of +60 mV, the current blockades of mTOR or FKBP12 were indistinguishable between their unbound and rapamycin-bound states (Figure S5). However, at a higher voltage of +80 mV, we observed distinct changes in current blockade upon the binding of rapamycin to mTOR or FKBP12 (Figure S6). This ability to discriminate protein–molecular glue interactions at higher voltages may be attributed to the stronger EOF within the YaxAB-C_9_ nanopore, which brings mTOR or FKBP12 closer to the constriction with a higher ion density and allows for a longer residence time within the pore. Thus, YaxAB-C_9_ nanopores can be effectively used to examine whether a specific molecular glue binds to its target protein, enabling the probing of protein–molecular glue interactions at the single-molecule level.

### Simultaneous Detection of Molecular Glue-Triggered Multiprotein States Using YaxAB Nanopores

**Figure 4:**
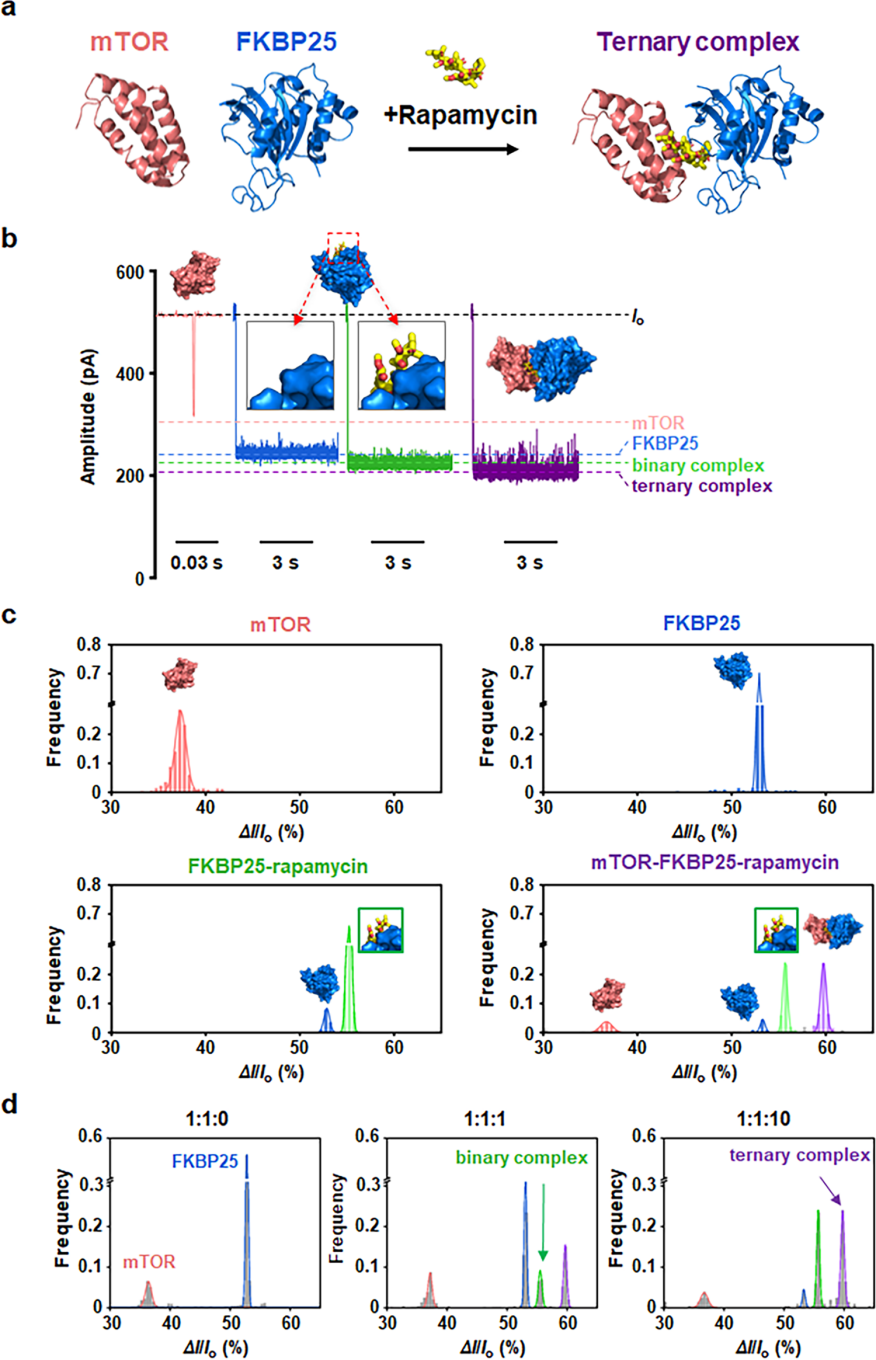
Simultaneous detection of single proteins, binary complex, and ternary complex using a YaxAB-C_9_ nanopore. (a) Formation of a rapamycin-induced ternary complex (mTOR-FKBP25-rapamycin). (b) Representative nanopore events showing differential blockade current signals corresponding to mTOR, FKBP25, FKBP25-rapamycin binary complex, and mTOR-FKBP25-rapamycin ternary complex. (c) Statistical analysis of histogram for Δ*I*/*I*_o_ corresponding to the detection of mTOR (top left), FKBP25 (top right), FKBP25-rapamycin (1:10) binary complex (bottom left), and mTOR-FKBP25-rapamycin (1:1:10) ternary complex (bottom right). (d) Statistical analysis for concentration-dependent nanopore experiments at 1:1:0, 1:1:1, and 1:1:10 molar ratios of mTOR, FKBP25, and rapamycin, respectively. Salmon, blue, green, and purple correspond to the nanopore event signals for mTOR, FKBP25, the FKBP25-rapamycin binary complex, and the mTOR-FKBP25-rapamycin ternary complex, respectively. All the electrical measurements were performed at an applied potential of +60 mV. Current traces were filtered with a Bessel (8-pole) filter at 1 kHz.

In contrast to most biological nanopores with fixed pore sizes, the funnel-shaped, size-adjustable structure of YaxAB nanopores offers the potential to accommodate folded proteins across a broader mass range. To test the ability of YaxAB-C_9_ nanopores to measure larger-sized proteins, we used the 25 kDa FK506-binding protein (FKBP25) (25.2 kDa, +7.0e) (Figure S7), which also forms a ternary complex (∼38.4 kDa) with mTOR through rapamycin (Figure 4a). First, we independently measured mTOR, FKBP25, the FKBP25-rapamycin mixture, and the mTOR-FKBP25-rapamycin mixture using YaxAB-C_9_ nanopores at an applied voltage of +60 mV. The current blockades corresponding to multiprotein states (mTOR, FKBP25, binary complex (FKBP25-rapamycin), and ternary complex (mTOR-FKBP25-rapamycin)) were clearly distinguishable using the YaxAB-C_9_ nanopore (Figure 4b). For positively charged proteins dragged by EPF and EOF with the same direction, they could be moved to a similar site near the constriction, where current blockade is likely to be mainly affected by molecular size: the larger-sized mTOR-FKBP25-rapamycin ternary complex exhibited higher current blockade than FKBP25. In the statistical histograms, the mean Δ*I*/*I*_o_ values for free mTOR, free FKBP25, binary, and ternary complexes were 37.30 ± 0.34, 52.75 ± 0.21, 55.24 ± 0.10, and 59.49 ± 0.23%, respectively (Figures 4c and S8). The current blockades of free FKBP25 and the FKBP25-rapamycin binary complex were unambiguously discriminated against a mixture of FKBP25 and rapamycin. Notably, the distinctive current blockade signals corresponding to a broad range of protein analytes mTOR (∼12 kDa), FKBP25 (∼25 kDa), the binary complex (∼26 kDa), and the ternary complex (∼38 kDa) were simultaneously observed in the mTOR-FKBP25-rapamycin mixture using a single YaxAB-C_9_ nanopore (Figure 4c, bottom right).

Next, we conducted additional nanopore experiments to assess the rapamycin dose-dependent formation of ternary complexes (Figures 4d and S9). Initially, at a 1:1:0 molar ratio of mTOR:FKBP25:rapamycin, only signals corresponding to mTOR and FKBP25 were observed. At a 1:1:1 molar ratio, current blockade signals from both the FKBP25-rapamycin binary complex and the mTOR-FKBP25-rapamycin ternary complex appeared. As the molar ratio of rapamycin increased to 1:1:10, the event populations of the binary and ternary complexes increased substantially, while those of the unbound individual proteins decreased (Figure 4d). Collectively, the YaxAB-C_9_ nanopore could simultaneously detect distinct multiprotein states induced by molecular glues, demonstrating its potential for efficient screening and single-molecule-level quantification of both binary and ternary complexes.

To evaluate protein–molecular glue interactions at lower analyte concentrations, we conducted the same nanopore experiments using a protein concentration of 10 nM. Even at this low concentration, single proteins, binary complexes, and ternary complexes were clearly detected (Figure S10). This demonstrates that YaxAB-C_9_ nanopores can identify molecular glue-induced protein complexes at picomolar levels, requiring approximately 6300 times less sample than what is needed for NMR. This capability could be highly advantageous for studying poorly soluble targets or small-molecule compounds, as well as for high-throughput applications that require only minimal sample quantities.

### Specific Detection of a Molecular Glue from Multiple Compounds Using YaxAB Nanopores

**Figure 5:**
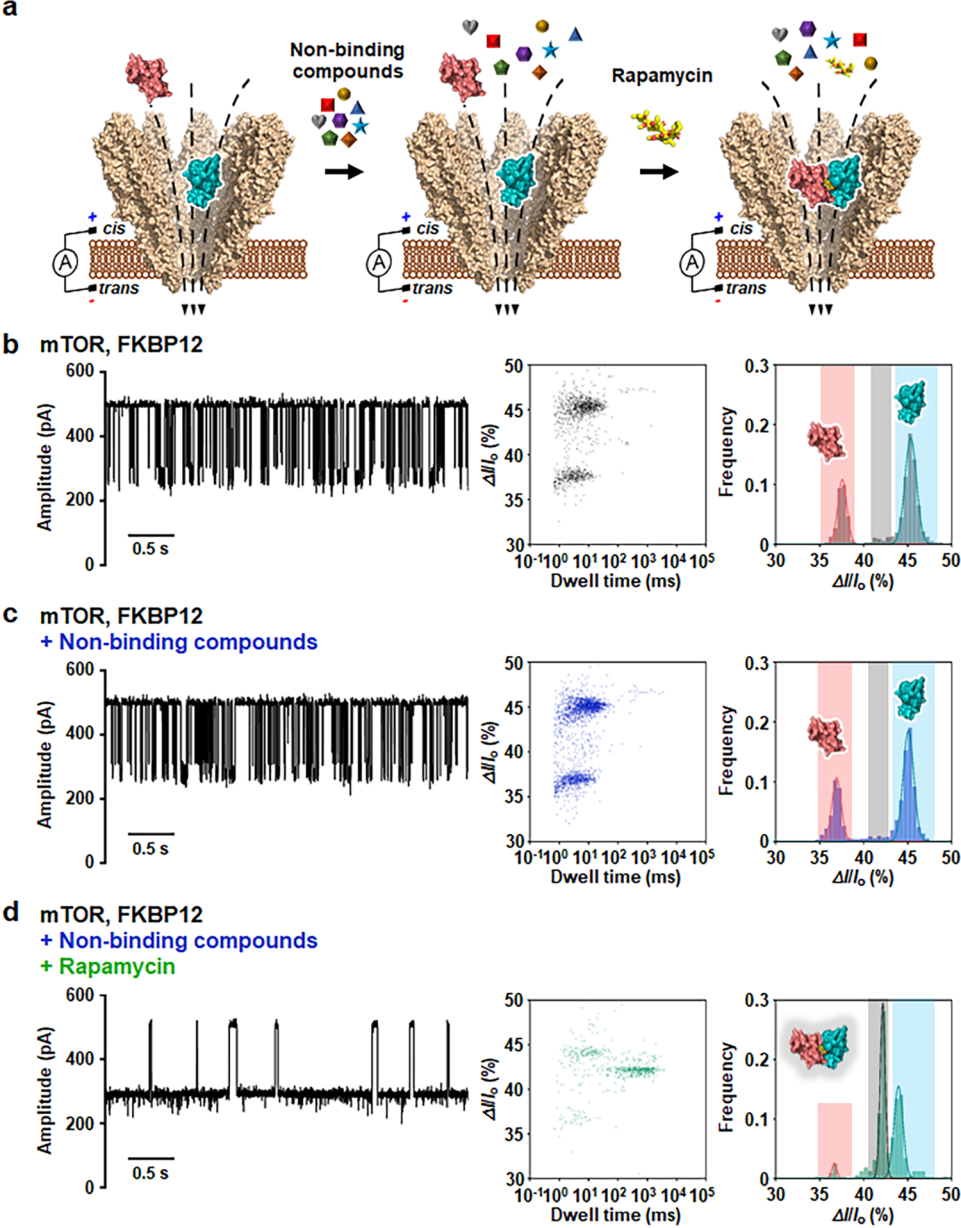
Specific detection of rapamycin from a mixture of compounds. (a) Schematic illustration of molecular glue detection using a YaxAB-C_9_ nanopore. The colored shapes symbolize nonbinding compounds (astemizole, terfenadine, capecitabine, sulfanilamide, mibefradil, pranlukast, dimantine, and monobenzone). (b–d) Representative current traces and statistical analysis of 2D scatter plots (Δ*I*/*I*_o_ versus dwell time) and statistical histograms corresponding to the detection of mTOR, FKBP12 (b), and mTOR, FKBP12 with a mixture of nonbinding compounds in the absence (c) or presence (d) of rapamycin. All of the small-molecule compounds were treated at a concentration of 1.6 μM to the *cis* side of a YaxAB-C_9_ nanopore. All of the electrical measurements were performed at an applied potential of +60 mV. Current traces were filtered with a Bessel (8-pole) filter at 1 kHz.

To determine whether this nanopore detection could be applied to screening of molecular glues from multiple compounds, we carried out YaxAB-C_9_ nanopore experiments with a mixture of multiple nonbinding compounds (astemizole, terfenadine, capecitabine, sulfanilamide, mibefradil, pranlukast, dimantine, and monobenzone) in the absence or presence of rapamycin (Figure 5a). The nonbinding activities of these compounds were confirmed by 1D NMR experiments (Figure S11). In the nanopore measurements, no change in the open conductance of the nanopore was observed when rapamycin alone or the multiple compounds were added without proteins (Figure S12), indicating that the nanopore’s sensing capabilities were not affected by the presence of rapamycin or multiple small-molecule compounds. When the mixture of nonbinding compounds was added to the YaxAB-C_9_ nanopore with mTOR and FKBP12, only the current blockade signals corresponding to the individual proteins were detected (Figure 5b,c). In contrast, when the mixture of nonbinding compounds with rapamycin was added to a YaxAB-C_9_ nanopore, the current blockade signals from the mTOR-FKBP12-rapamycin ternary complex predominated, as confirmed by statistical analysis (Figure 5d). These results demonstrate that YaxAB-C_9_ nanopores can specifically identify hit-molecular glues from a mixture of compounds, highlighting their potential for screening molecular glues against a library of small-molecule compounds. Although our finding is useful for the mixture-based screening approach as employed by NMR,^35^ efficient molecular glue screening for practical application awaits the integration of a nanopore array with high-throughput sensing through nano/microfluidics.

### Single-Molecule Fingerprinting of Molecular Glues for Binary Protein Interaction Using YaxAB Nanopores

**Figure 6:**
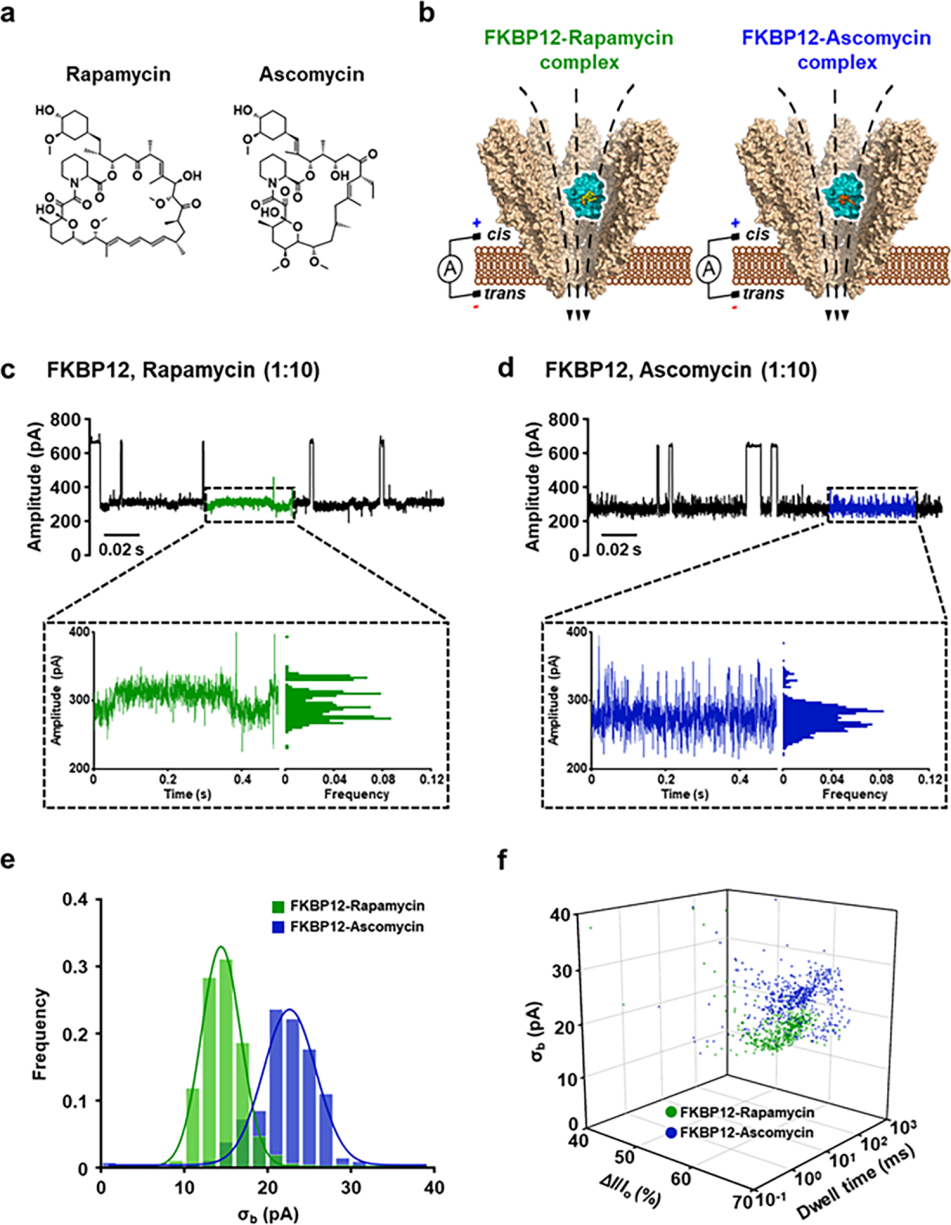
Discrimination between rapamycin- and ascomycin-bound FKBP12 complexes using YaxAB nanopores. (a) Chemical structural formula of rapamycin and ascomycin. (b) Schematic illustration of the detection of the rapamycin- and ascomycin-induced binary complexes. (c, d) Representative current traces and statistical analysis of current histograms corresponding to the detection of FKBP12-rapamycin complex (c) and the detection of FKBP12-ascomycin complex (d). (e) Histograms of σ_b_ for the binary complexes. FKBP12-rapamycin (σ_b_ = 15.0 ± 0.8 pA) and FKBP12-ascomycin (σ_b_ = 22.1 ± 0.5 pA) are shown in green and blue, respectively. (f) 3D scatter plot based on Δ*I*/*I*_o_, log dwell time, and σ_b_ for the binary complexes. The three features can assign the blockades of binary complexes into two distinct rapamycin and ascomycin groups. All electrical measurements were performed at an applied potential of +80 mV. Current traces were filtered with a Bessel (8-pole) filter at 1 kHz. The proteins and molecular glues were treated to the *cis* side of a YaxAB nanopore at a 1:10 ratio.

Monitoring binary interactions between molecular glues and target proteins is important for understanding binding stoichiometry, mechanism of action, and optimizing their efficacy.^36^ To explore the potential for single-molecule fingerprinting of protein–molecular glue interactions, we examined another molecular glue, ascomycin (FK520), a natural analogue of FK506. Ascomycin binds to FKBP12 but forms a ternary complex with calcineurin, not with mTOR.^37^ Ascomycin and rapamycin share a common tricycle backbone necessary for binding to FKBPs (Figure 6a), but differ in the polyketide backbone required for binding to calcineurin or mTOR.^38^ At an applied potential of +80 mV and a 1:10 molar ratio of FKBP12 to rapamycin or ascomycin, we observed significant differences in current blockade between FKBP12-rapamycin and FKBP12-ascomycin binary complexes (Figure 6b–d). Thus, the YaxAB-C_9_ nanopores enabled us to distinguish between two distinct molecular glues bound to the same protein at the single-molecule level. Particularly, even the subtle size difference (∼122 Da) between ascomycin (M.W., 792.01) and rapamycin (M.W., 914.18) could be discriminated. The standard deviation of current blockade fluctuations (σ_b_) was also used to profile the blockage characteristics of single molecules.^39^ The statistical histogram of σ_b_ for the binary complexes showed an excellent distinction between them (Figure 6e). With a combination of Δ*I*/*I*_o_, log dwell time, and σ_b_ features, the three-dimensional (3D) scatter plot revealed two distinguishable populations of binary complexes (Figure 6f). The noticeable changes in current blockade levels and fluctuations induced by different molecular glues suggest that YaxAB-C_9_ nanopores can be used for single-molecule fingerprinting of molecular glues in binary protein interactions.

### Single-Molecule Fingerprinting of Molecular Glues for Ternary Protein Interaction Using YaxAB Nanopores

**Figure 7:**
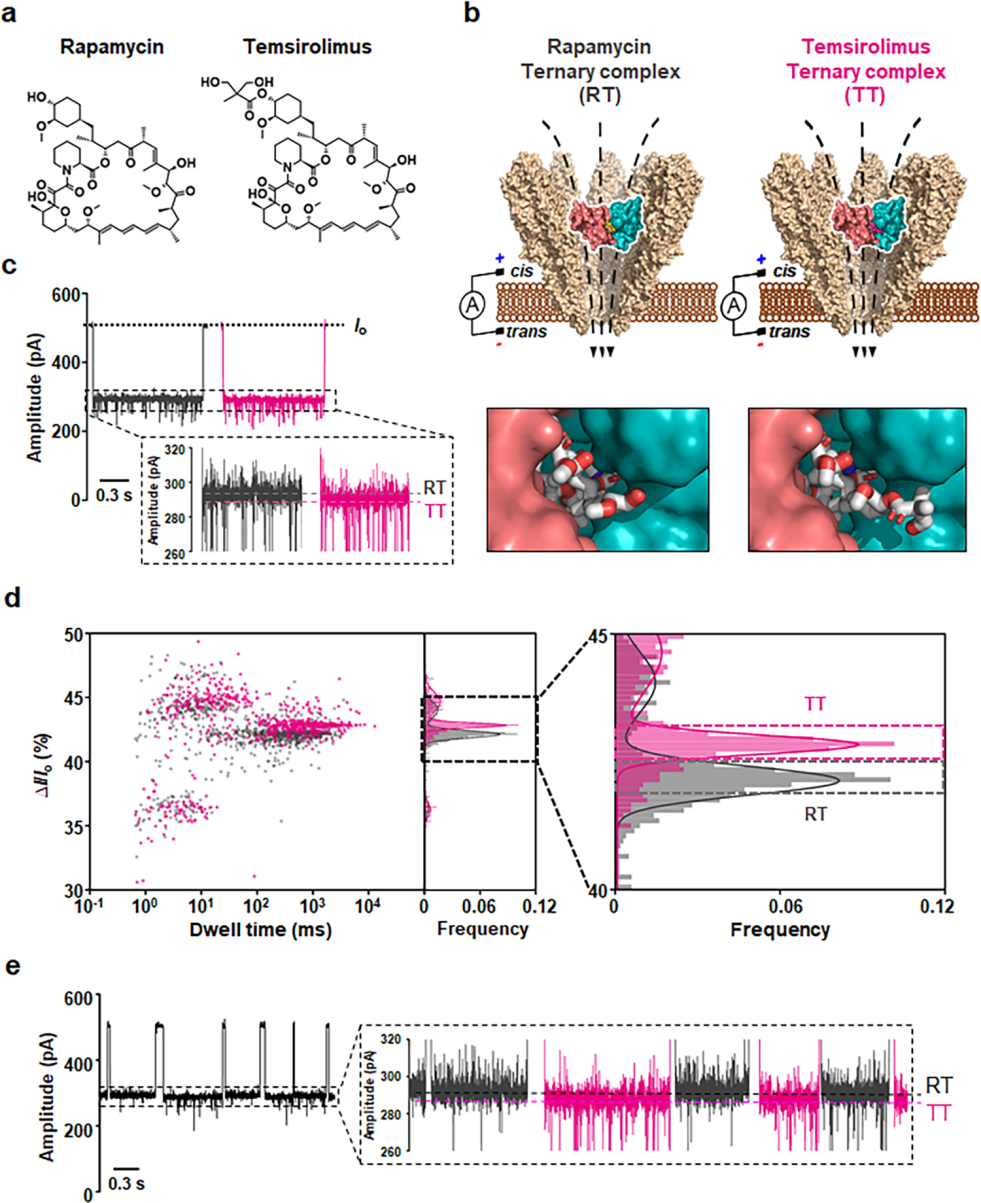
Discrimination between rapamycin- and temsirolimus-bound ternary complexes using YaxAB nanopores. (a) Chemical structural formula of rapamycin and temsirolimus. (b) Schematic illustration of the detection of the rapamycin-induced ternary complex (RT) and the temsirolimus-induced ternary complex (TT) with the binding interfaces of the ternary complexes formed by rapamycin (bottom left) and temsirolimus (bottom right). The terminal part of temsirolimus was projected toward the outside of the proteins, unlike that of rapamycin. The structure of the ternary complex by rapamycin was obtained from the protein data bank (1FAP), and the structure of the ternary complex by temsirolimus obtained by docking with AutoDock Vina 1.1.2. (c) Representative event signals consisting of the blockade current and dwell time corresponding to RT and TT. (d) Overlaid 2D scatter plots (Δ*I*/*I*_o_ versus dwell time) corresponding to the detection of RT (black) and TT (magenta) at the 1:1:5 molar ratio (mTOR:FKBP12:molecular glue). (e) Representative current trace corresponding to the simultaneous detection of RT (black) and TT (magenta). Both molecular glues were premixed with the mTOR and FKBP12 at the 1:1:0.5:0.5 molar ratio (mTOR:FKBP12:rapamycin:temsirolimus). All the electrical measurements were performed at an applied potential of +60 mV. Current traces were filtered with a Bessel (8-pole) filter at 1 kHz.

For applicability to structure–activity relationship (SAR) analysis and lead optimization, it is essential to distinguish ternary complexes formed by structurally similar molecular glues. To explore the potential for single-molecule fingerprinting of molecular glues in ternary protein interactions, we conducted nanopore experiments with a rapamycin derivative, temsirolimus. Similar to rapamycin, temsirolimus forms a ternary complex with mTOR and FKBP12 (Figure 7b). Temsirolimus, a soluble ester of rapamycin, reduces immunosuppressive effects while enhancing pharmacokinetic properties.^40^ Although rapamycin and temsirolimus share a central macrolide structure, they differ in the functional groups at C_40_, with temsirolimus featuring a dihydroxymethyl propionic acid ester group at this position (Figure 7a).^41^ To determine whether YaxAB-C_9_ nanopores can discriminate between rapamycin- and temsirolimus-bound ternary complexes, we performed nanopore measurements with both complexes. Notably, we observed distinct differences in current blockades upon the addition of rapamycin or temsirolimus to mTOR and FKBP12 (Figure 7c). Interestingly, overlaid 2D scatter plots revealed that the event distribution for the temsirolimus-induced ternary complex (mean Δ*I*/*I*_o_ value = 42.74 ± 0.08%) was clearly distinguishable from that of the rapamycin-induced ternary complex (mean Δ*I*/*I*_o_ value = 42.15 ± 0.03%) (Figure 7d).

To further confirm that the observed nanopore events originated from the ternary complex induced by temsirolimus, we conducted additional nanopore experiments in which temsirolimus was titrated into a mixture of mTOR, FKBP12, and rapamycin. In this setup, we observed two distinct types of ternary complex signals simultaneously, indicating that current blockades could differentiate between the rapamycin-induced ternary complex (RT) and the temsirolimus-induced ternary complex (TT) using a single YaxAB-C_9_ nanopore (Figure 7e). As the concentration of temsirolimus increased from 0 to 80 nM, the event populations of TT gradually increased, confirming that the observed nanopore events were indeed the result of temsirolimus-induced PPI (Figure S13).

The current blockade distinction between RT and TT detected by YaxAB nanopores may arise from the difference in molecular size (rapamycin M.W., 914.18 Da; temsirolimus M.W., 1030.29 Da). In the TT structure, the dihydroxymethyl propionic acid ester group of temsirolimus is exposed on the exterior of the ternary complex (Figure 7b). Notably, the YaxAB nanopores were able to sensitively discriminate even a subtle size difference of ∼116 Da in molecular size between RT and TT, which accounts for 0.46% of the total ternary complex mass. This high-resolution discrimination capability could be harnessed to identify compounds with stronger binding activities, suggesting the potential for a single-molecule-based “SAR-by-nanopore” approach for molecular glues. Taken together, we present a proof-of-concept for single-molecule fingerprinting of molecular glues in ternary protein interactions using YaxAB nanopores, offering a valuable tool for single-molecule-based SAR analysis of molecular glues for lead optimization and mode-of-action (MOA) studies.

## Conclusions

In current drug discovery, bifunctional small-molecules, such as molecular glues and proteolysis-targeting chimeras (PROTACs), are promising drug modalities due to their potential to target otherwise “hard-to-drug” (or “undruggable”) disease targets. However, the broad-spectrum screening of small molecules for molecular glues and PROTACs has been hampered by the current limitations of conventional ensemble-averaging-based techniques. Cell-based and in vitro assays, including TR-FRET, NanoBRET, SPR, and NMR, often require time- and cost-consuming labeling process, which would often result in inaccurate evaluations, such as false positives. Due to their single-molecule sensing capabilities, YaxAB nanopores offer significant advantages over conventional approaches for screening molecular glues: (1) label-free detection and single-molecule-based protein quantification, enabling highly accurate evaluation of molecular glue efficacy; (2) picomole-scale detection with approximately 6300-fold higher sensitivity than NMR, which is particularly beneficial for poorly soluble targets and compounds; and (3) ultrahigh resolution, allowing for the sensitive discrimination of subtle molecular size differences, such as those between binary complexes formed by rapamycin and ascomycin (∼122 Da) or between ternary complexes formed by rapamycin and temsirolimus (∼116 Da). This single-molecule fingerprinting of molecular glues could lead to the development of a “SAR-by-nanopore” approach, contributing to the rational design of molecular glues for lead optimization. Taken together, we propose that YaxAB nanopores serve as a valuable platform for label-free, ultrasensitive, and high-resolution screening and SAR analysis of molecular glues. In the future, nanopore-based monitoring of the protein-molecular glue binding process, even in a single event, could enable single-molecule-level observation of a conformational change of molecular glues, which is accompanied by recruitment of neo-substrate. This will contribute to the molecular understanding of mechanism of action and therapeutic efficacies of molecular glues. Furthermore, integrating a nanopore array with high-throughput sensing and artificial intelligence (AI)-based data analysis could facilitate the efficient discovery of molecular glues through high-throughput screening (HTS).

In this study, we introduced a YaxAB nanopore sensor capable of monitoring molecular glue-triggered protein complex formation at the single-molecule level. The funnel-shaped, size-adjustable structure and nanofluidic properties of YaxAB nanopores enabled the electro-osmotic trapping of a wide range of proteins and allowed single-molecule analysis of multiprotein states induced by molecular glues (e.g., mTOR, FKBPs, FKBPs-rapamycin, and mTOR-FKBPs-rapamycin). Notably, a single YaxAB nanopore could simultaneously detect both binary and ternary complexes, allowing for the highly accurate, quantitative evaluation of molecular glue efficacy. Furthermore, YaxAB nanopores demonstrated the capability to sensitively discriminate between molecular glues bound to the same proteins and protein complexes, suggesting the potential for single-molecule fingerprinting of molecular glues in binary and ternary protein interactions. Taken together, our findings provide a proof-of-concept that YaxAB nanopores can be used as a label-free, ultrasensitive, and high-resolution platform, particularly suited for the screening and SAR analysis of molecular glues at the single-molecule level. This advancement will contribute to low-cost, highly efficient discovery and rational design of bifunctional drug modalities, including molecular glues and PROTACs.

## Methods

### Chemicals

Chemicals were purchased from Sigma-Aldrich (St. Louis, MO), unless otherwise specified. The small-molecule drugs rapamycin (914.18 g/mol, Selleckchem, Houston), ascomycin (792.01 g/mol, Selleckchem, Houston), temsirolimus (1030.29 g/mol, Selleckchem, Houston), astemizole (458.57 g/mol, MedChemExpress, New Jersey), terfenadine (471.67 g/mol, MedChemExpress), capecitabine (359.35 g/mol, MedChemExpress), sulfanilamide (172.20 g/mol, MedChemExpress), mibefradil (568.55 g/mol, MedChemExpress), pranlukast (481.50 g/mol, MedChemExpress), dimantine (297.56 g/mol, Biosynth, Staad, Germany), and monobenzone (200.23 g/mol, MedChemExpress) were dissolved in dimethyl sulfoxide (DMSO).

### Purification of YaxAB Nanopores and Proteins

YaxA and YaxB were expressed and purified, and YaxAB-C_9_ pore complexes were generated as previously described.^21,28^ mTOR (PDB ID: 1FAP) and the mTOR_F2108L mutant were expressed and purified following established protocols.^42^ Briefly, the proteins were expressed in BL21(DE3) *Escherichia coli* cells induced with 0.5 mM isopropyl-β-d-thiogalactoside (IPTG). Cell pellets were resuspended and sonicated to disrupt the cells. The supernatant was loaded onto a GSTrap HP column (Cytiva, Chicago, IL) and eluted with 10 mM glutathione. Fractions containing the target proteins were dialyzed and incubated with thrombin protease to cleave the GST tag from the target proteins. The GST tag was subsequently removed using a GSTrap HP column. The digested proteins were further purified using a HiLoad 16/600 Superdex 75pg column (Cytiva, Chicago, IL). FKBP12 (PDB ID: 1FAP) was expressed and purified as previously described.^43^ Briefly, FKBP12 was expressed in BL21(DE3) *E. coli* cells induced with 0.5 mM IPTG. Harvested cell pellets were resuspended and sonicated to disrupt the cells. The supernatant was loaded onto a HisTrap HP column (Cytiva, Chicago, IL) and eluted using a linear gradient from 10 mM to 1 M imidazole. FKBP12 proteins were further purified using a HiLoad 16/600 Superdex 75pg column. FKBP25 (PDB: 2MPH) was expressed and purified as previously described.^44^ Briefly, FKBP25 was expressed in BL21(DE3) *E. coli* cells induced with 0.2 mM IPTG. After being harvested, the cell pellets were resuspended and sonicated. The supernatant was loaded onto a HisTrap HP column and eluted using a linear gradient up to 1 M imidazole. Fractions containing the target proteins were dialyzed and incubated with SUMO protease to cleave the 6x-His-SUMO tag, which was then removed using a HisTrap HP column. The target protein fractions were further purified by loading them onto a HiTrap SP column (Cytiva, Chicago, IL) and eluted using a linear gradient up to 1 M NaCl. FKBP25 proteins were further purified using a HiLoad 16/600 Superdex 75pg column.

### NMR Spectroscopy

All 1D and 2D NMR experiments were conducted using 700 and 800 MHz NMR spectrometers (Bruker) equipped with cryogenic probes at the Korea Basic Science Institute (KBSI, Ochang, Republic of Korea). The 1D NMR experiments of free small-molecule compounds and their complexes with nonlabeled mTOR protein were performed in a buffer containing 50 mM sodium phosphate (pH 6.5) and 50 mM sodium chloride. The 2D NMR samples were prepared in 90% H_2_O/10% D_2_O with 50 mM sodium phosphate (pH 6.5) and 50 mM sodium chloride. The 2D ^1^H–^15^N HSQC NMR experiments were conducted at 25 °C using 0.1 mM ^15^N-labeled mTOR in the absence or presence of FKBP12, rapamycin, and FKBP12/rapamycin in equal ratios. All NMR data were processed and analyzed using TopSpin 3.6.2, NMRPipe/NMRDraw, and SPARKY software.

### MD Simulations

To characterize the YaxAB-C_9_ nanopore using MD simulations, we constructed a simulation setup with a YaxAB-C_9_ nanopore embedded in a lipid membrane. We prepared a lipid bilayer system that measured approximately 27 × 27 nm^2^ using Membrane Builder^45^ in CHARMM-GUI.^46^ We immersed the complex system of the lipid membrane and nanopores in a solution of 1 M KCl. Then, we performed MD simulations using the GROMACS 2022.4 package.^47^ We employed the CHARMM36m force field^48^ combined with the CHARMM-modified TIP3P model. To enhance charge–charge interaction pairs, we applied the CUFIX corrections to the CHARMM36m force field set.^49^ The model structure was first minimized with steepest descent followed by maximum force under 100 kJ mol^–1^ nm^–1^. We performed the packing of lipid membrane under a 50 ns constant surface tension-constant temperature (NPγT) ensemble: surface tension was zero (γ = 0)^50^ and the temperature was 303 K.^51^ For the computation of van der Waals forces, we employed a 10 to 12 Å switching scheme. We computed the long-range electrostatic forces using the particle-mesh Ewald summation scheme^52^ with a 1.2 Å grid spacing and a 12 Å real-space cutoff. Covalent bonds to hydrogen in nonwater and water molecules were constrained using the LINCS^53^ and SETTLE^54^ algorithms. The ion or water flux simulations performed a 160 ns NVT ensemble under the applied voltage (+60 mV). The average ion flux was then computed over the last 110 ns of the simulations. We visualized the density-flux map using the method described previously.^55^

### Single-Channel Measurements and Recordings

The two compartments of the chamber were separated by a poly(tetrafluoroethylene) (PTFE) film (Goodfellow Cambridge Limited, Huntingdon, England) containing a 100 μm aperture.^56^ A planar lipid bilayer was formed on an aperture prepainted with 3.0% (w/v) of lipid stock. Two compartments were filled with 800 μL of electrolyte buffer (10 mM Tris–HCl pH 8.0, 1 mM EDTA, 1 M KCl). The ground Ag/AgCl electrode was connected to the *trans* compartment, while the working Ag/AgCl electrode was connected to the *cis* compartment to measure current in the nanopore system. A small amount of YaxAB-C_9_ pore samples was added to the *cis* compartment, and the pore was inserted into the lipid bilayer formed on the aperture. Upon successful insertion of a stable YaxAB-C_9_ nanopore, proteins (mTOR, mTOR_F2108L mutant, FKBP12, and FKBP25) at 160 nM and small-molecule compounds (rapamycin, temsirolimus, astemizole, terfenadine, capecitabine, sulfanilamide, mibefradil, pranlukast, dimantine, and monobenzone) at concentrations ranging from 0.08 to 1.6 μM were introduced into the *cis* compartment. These were then detected under applied potentials of +60 and +80 mV by using the YaxAB-C_9_ nanopore. All electrophysiological measurements were conducted using a patch-clamp amplifier system (Axopatch 200B, Molecular Devices Inc., Sunnyvale, CA) and a Digidata 1550B A/D converter (Molecular Devices Inc., Sunnyvale, CA).^57^ Measurement data were recorded using Clampex 11.2 software (Molecular Devices Inc., Sunnyvale, CA) at sampling rates of 100 kHz and with a low-pass Bessel (8-pole) filter at 10 kHz. All nanopore experiments were conducted at room temperature.

### Nanopore Data Analysis

All nanopore data were analyzed using Clampfit 11.2 software (Molecular Devices Inc., Sunnyvale, CA). For data processing, all current blockade events were additionally filtered using a low-pass Bessel (8-pole) at 1 kHz except for the data shown in Figure S6a,b. Current blockade was defined as Δ*I*/*I*_o_, where Δ*I* is the blockade current and *I*_0_ is the open pore current. The mean values of current blockade and dwell time were obtained by fitting the histograms with a Gaussian function and a single exponential decay function, respectively. All values, including error bars, were derived from at least three independent nanopore experiments. The fraction of ternary complexes (mTOR-FKBP12-rapamycin) was calculated using eq 1
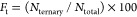
1where *F*_t_ is the fraction of ternary complex, *N*_ternary_ is the number of events for ternary complexes, and *N*_total_ is the total number of events for nanopore measurements. The fraction of ternary complexes was determined based on the three independent nanopore measurements.

### Measurement of Ion Selectivity of YaxAB-C_9_ Nanopore

To determinate ion selectivity of the YaxAB-C_9_ nanopore under pH 8.0 conditions, the ion selectivity was calculated using the Goldman-Hodgkin-Katz equation (eq 2)
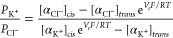
2where [α_K^+^/Cl^–^_]_*cis*/*trans*_ is the activity of K^+^ or Cl^–^ ions in the *cis* or *trans* compartments, *R* is the gas constant, *T* is the temperature, *F* is the Faraday’s constant, and *V*_r_ is the reversal potential. *V*_r_ was measured under asymmetric salt conditions. A single YaxAB-C_9_ nanopore was first inserted into a lipid bilayer under symmetric salt concentrations in both compartments (800 μL, 10 mM Tris–HCl pH 8.0, 1 mM EDTA, and 1 M KCl), with electrodes balanced. Then, 400 μL of the 1 M KCl electrolyte solution in both compartments was exchanged with 400 μL of 3 M KCl solution and 0 M KCl solution in the *trans* and *cis* compartments, respectively. Ion activity was calculated using the mean ion activity coefficients (0.573 and 0.649 for 2 and 0.5 M KCl, respectively).^58^ The solutions were gently mixed, and *I*–*V* curves were recorded to determine the reversal potentials.

## Supporting Information Available

2D ^1^H–^15^N HSQC spectra for rapamycin-induced interaction between mTOR and FKBP12; properties and size dimensions of protein analytes; voltage-dependent nanopore experiments of mTOR and FKBP12; nanopore measurements of single proteins (mTOR and FKBPs) and molecular glue-induced binary and ternary complexes; 1D CPMG NMR spectra of multiple compounds; nanopore measurements of rapamycin or a mixture of small-molecule compounds without protein; statistical analysis of 2D scatter plots for rapamycin- and temsirolimus-induced ternary complexes (Figures S1–S13) (PDF)
