# Supplementary material for: Single-Molecule-Based, Label-Free Monitoring of Molecular Glue Efficacies for Promoting Protein–Protein Interactions Using YaxAB Nanopores
Source: ACS Nano. 2024 Nov 1;18(45):31451–65. doi: 10.1021/acsnano.4c11436 (PMC11562796; doi:10.1021/acsnano.4c11436)
Supplement: Supplementary file 1 — nn4c11436_si_001.pdf [file nn4c11436_si_001.pdf]

Supporting information

Single-Molecule-Based, Label-Free Monitoring of Molecular Glue Efficacies for Promoting Protein-Protein Interactions Using YaxAB Nanopores

*Minju Ryu[1,2,‡], Sohee Oh[1,‡], Ki-Baek Jeong[1,3,‡], Sungbo Hwang[1], Jin-Sik Kim[1,3], Minji Chung[1,2], Seung-Wook Chi[1,2,4]**

[1]Disease Target Structure Research Center, Division of Biomedical Research, Korea Research Institute of Bioscience and Biotechnology (KRIBB), Daejeon 34141, Republic of Korea.

[2]Department of Proteome Structural Biology, KRIBB School of Bioscience, University of Science and Technology, Daejeon 34113, Republic of Korea.

[3]Critical Diseases Diagnostics Convergence Research Center, KRIBB, Daejeon 34141, Republic of Korea.

[4]School of Pharmacy, Sungkyunkwan University, Suwon, Gyeonggi, 16419, Republic of Korea

[‡]Contributed equally to the work

*Corresponding author (Email: swchi@kribb.re.kr)

KEYWORDS: Biological nanopore, molecular glue, protein-protein interaction, single-molecule, label-free analysis, drug screening

Table of contents

Figure S1. Verification of rapamycin-induced interaction between mTOR and FKBP12 using NMR spectroscopy.

Figure S2. Properties and size dimensions of mTOR, FKBP12, and mTOR-FKBP12-rapamycin ternary complex.

Figure S3. Measurements of current signals from mTOR using YaxAB nanopores at various voltages.

Figure S4. Measurements of current signals from FKBP12 using YaxAB nanopores at various voltages.

Figure S5. Measurements of current signals from single proteins (mTOR and FKBP12) and binary complexes (mTOR-rapamycin and FKBP12-rapamycin).

Figure S6. Discrimination between single proteins and binary complexes.

Figure S7. Properties and size dimensions of FKBP25, FKBP25-rapamycin binary complex, and mTOR-FKBP25-rapamycin ternary complex.

Figure S8. Measurements of current signals from mTOR, FKBP25, FKBP25-rapamycin binary complex, and mTOR-FKBP25-rapamycin ternary complex.

Figure S9. Formation of rapamycin-dependent mTOR-FKBP25-rapamycin ternary complex.

Figure S10. YaxAB nanopore measurements of single proteins and binary and ternary complexes at a low protein concentration of analytes.

Figure S11. 1D CPMG NMR spectra for multiple compounds.

Figure S12. YaxAB nanopore measurements with rapamycin or a mixture of small molecule compounds.

Figure S13. Discrimination between rapamycin- and temsirolimus-induced ternary complexes.

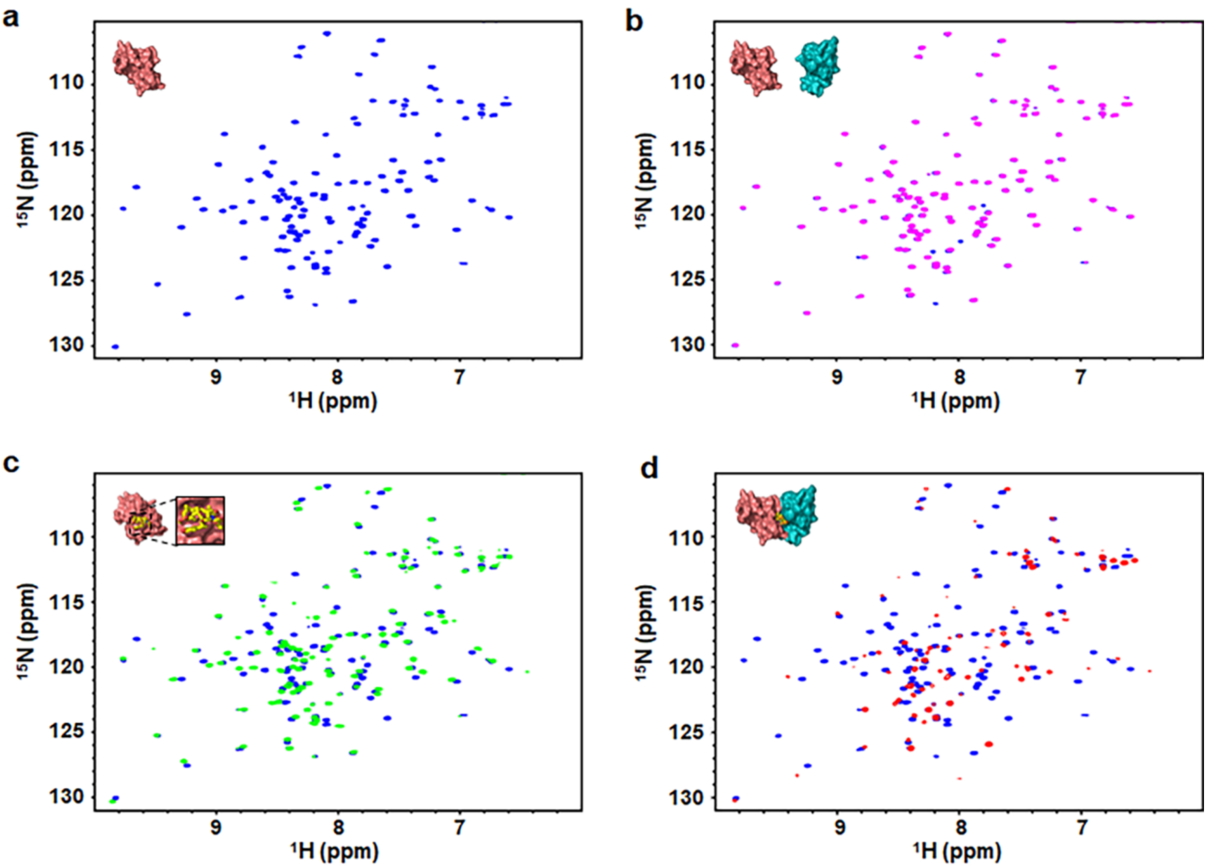

Figure S1. Verification of rapamycin-induced interaction between mTOR and FKBP12 using NMR spectroscopy. (a) The 2D ^{1}H-^{15}N HSQC full spectra of ^{15}N-labeled mTOR proteins (blue). (b) The overlaid ^{1}H-^{15}N HSQC spectra of the mTOR proteins in the absence (blue) or presence of FKBP12 (magenta) at a 1:1 ratio. (c) The HSQC spectra of the complex with mTOR and rapamycin (green) overlaid to that of free ^{15}N-labeled mTOR (blue) (molar ratio of 1:1) (d) The overlaid 2D HSQC spectra for the mTOR proteins (blue) and the ternary complex with mTOR, FKBP12 and rapamycin (red) at a molar ratio of 1:1:1.

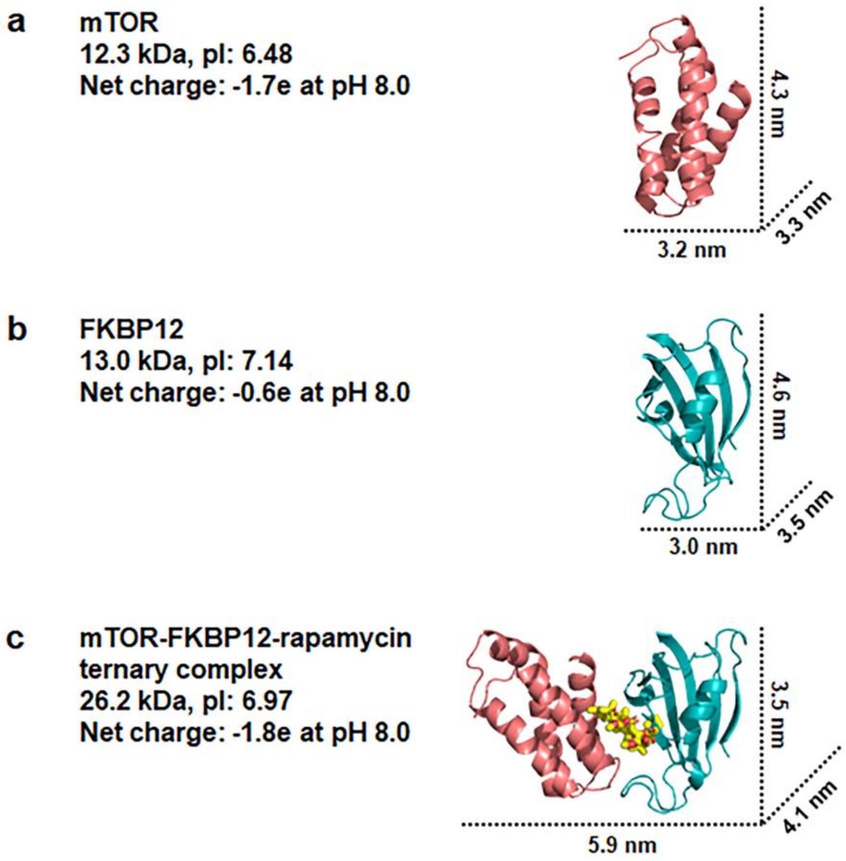

Figure S2. Properties and size dimensions of mTOR, FKBP12, and mTOR-FKBP12-rapamycin ternary complex. (a) mTOR, (b) FKBP12, and (c) mTOR-FKBP12-rapamycin ternary complex. The dimensions of the proteins were measured by executing the Draw_Protein_Dimensions.py script in Pymol.

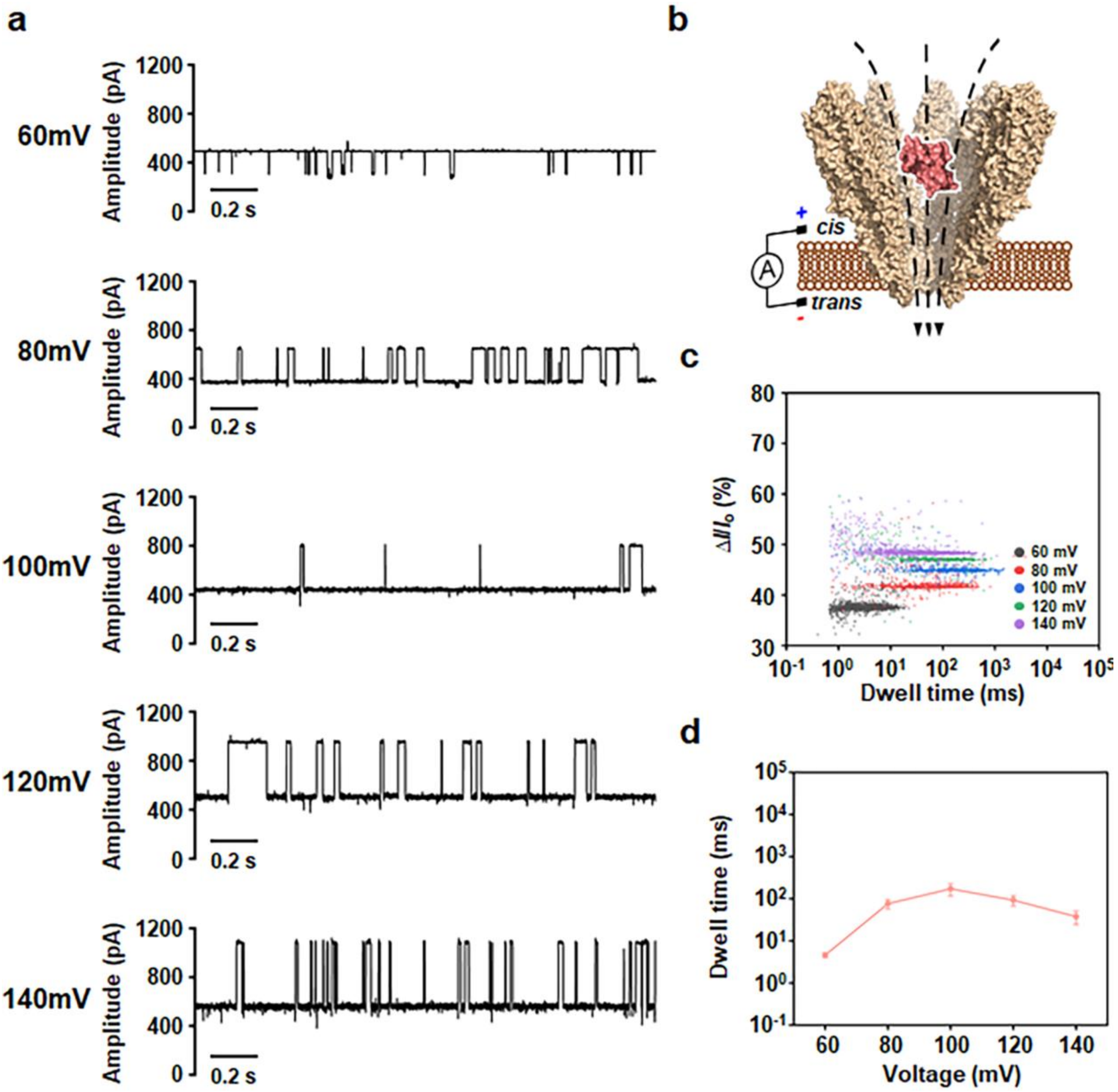

Figure S3. Measurements of current signals from mTOR using YaxAB nanopores at various voltages. (a) Representative current traces corresponding to the detection of mTOR at various voltages from +60 to +140 mV. (b) Schematic illustration of the detection of mTOR by using YaxAB nanopores. (c) Statistical analysis of the scatter plots ($\Delta I/I_o$ versus dwell time) corresponding to the detection of mTOR at various voltages. (d) Statistical analysis of the dwell times of mTOR at various voltages. mTOR translocates through the nanopore at applied potentials above +100 mV.

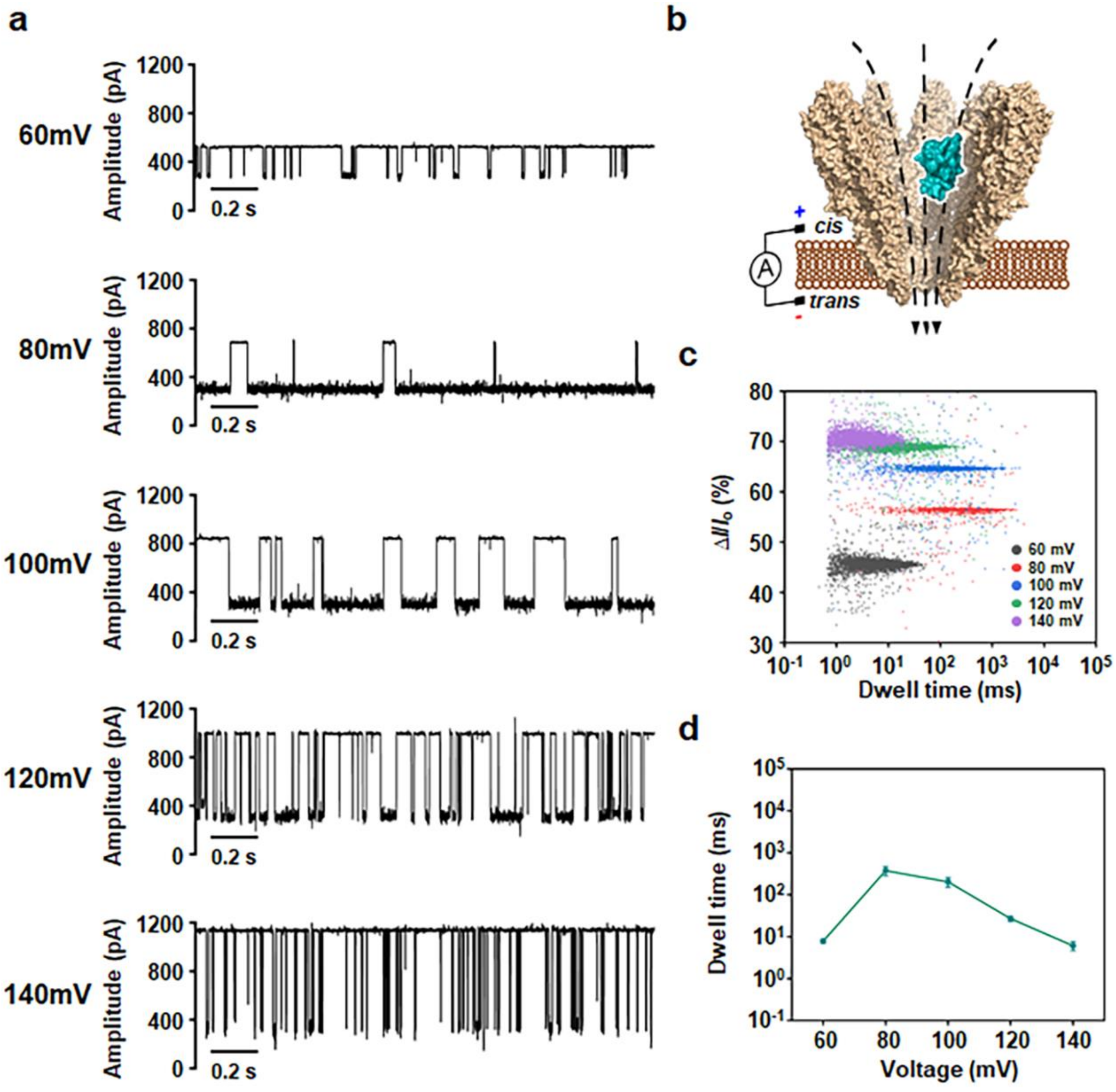

Figure S4. Measurements of current signals from FKBP12 using YaxAB nanopores at various voltages. (a) Representative current traces corresponding to the detection of FKBP12 at various voltages from +60 to +140 mV. (b) Schematic illustration of the detection of FKBP12 by using YaxAB nanopores. (c) Statistical analysis of the scatter plots ($\Delta I/I_o$ versus dwell time) corresponding to the detection of FKBP12 at various voltages. (d) Statistical analysis of the dwell times of FKBP12 at various voltages. FKBP12 translocates through the nanopore at applied potentials above +80 mV.

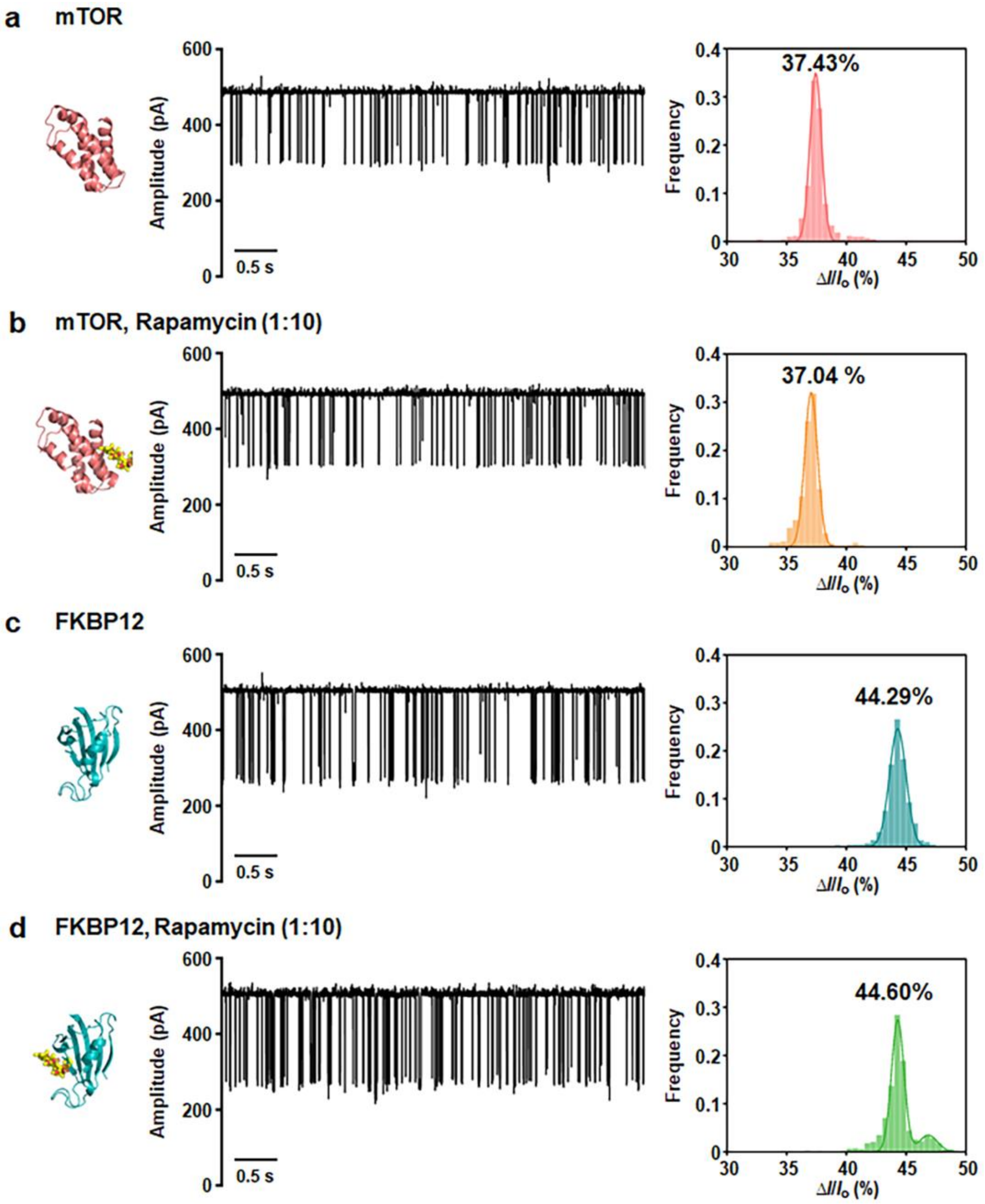

Figure S5. Measurements of current signals from single proteins (mTOR and FKBP12) and binary complexes (mTOR-rapamycin and FKBP12-rapamycin). (a-d) Representative current traces and statistical analysis of histograms ($\Delta I/I_o$) corresponding to the detection of mTOR in the absence (a) or presence of rapamycin (b), and FKBP12 in the absence (c) or presence of rapamycin (d). The proteins and rapamycin were treated to the *cis* side of YaxAB nanopores at a 1:0 (a,c) and 1:10 (b,d), ratios, respectively. All the electrical measurements were performed at an applied voltage of +60 mV.

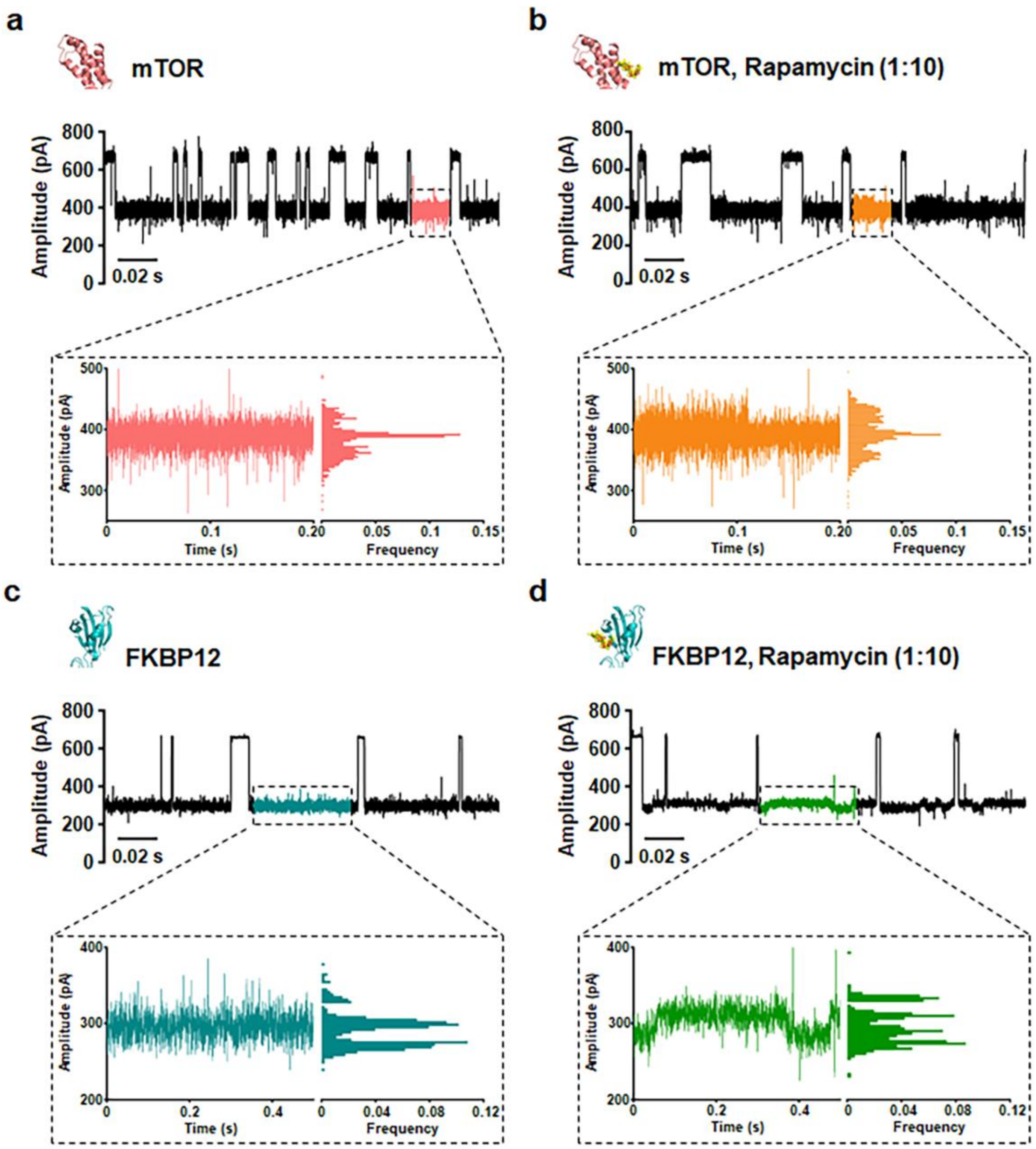

Figure S6. Discrimination between single proteins and binary complexes. (a-d) Representative current traces and statistical analysis of current histograms corresponding to the detection of mTOR in the absence (a) and presence of rapamycin (b), and FKBP12 in the absence (c) and presence (d) of rapamycin. Current traces were filtered with a Bessel (8-pole) filter at 10 (a-b) and 1 kHz (c-d). The proteins and rapamycin were treated to the *cis* side of YaxAB nanopores at a 1:0 ratio (a, c) and 1:10 ratio (b, d), respectively. All the electrical measurements were performed at an applied voltage of +80 mV.

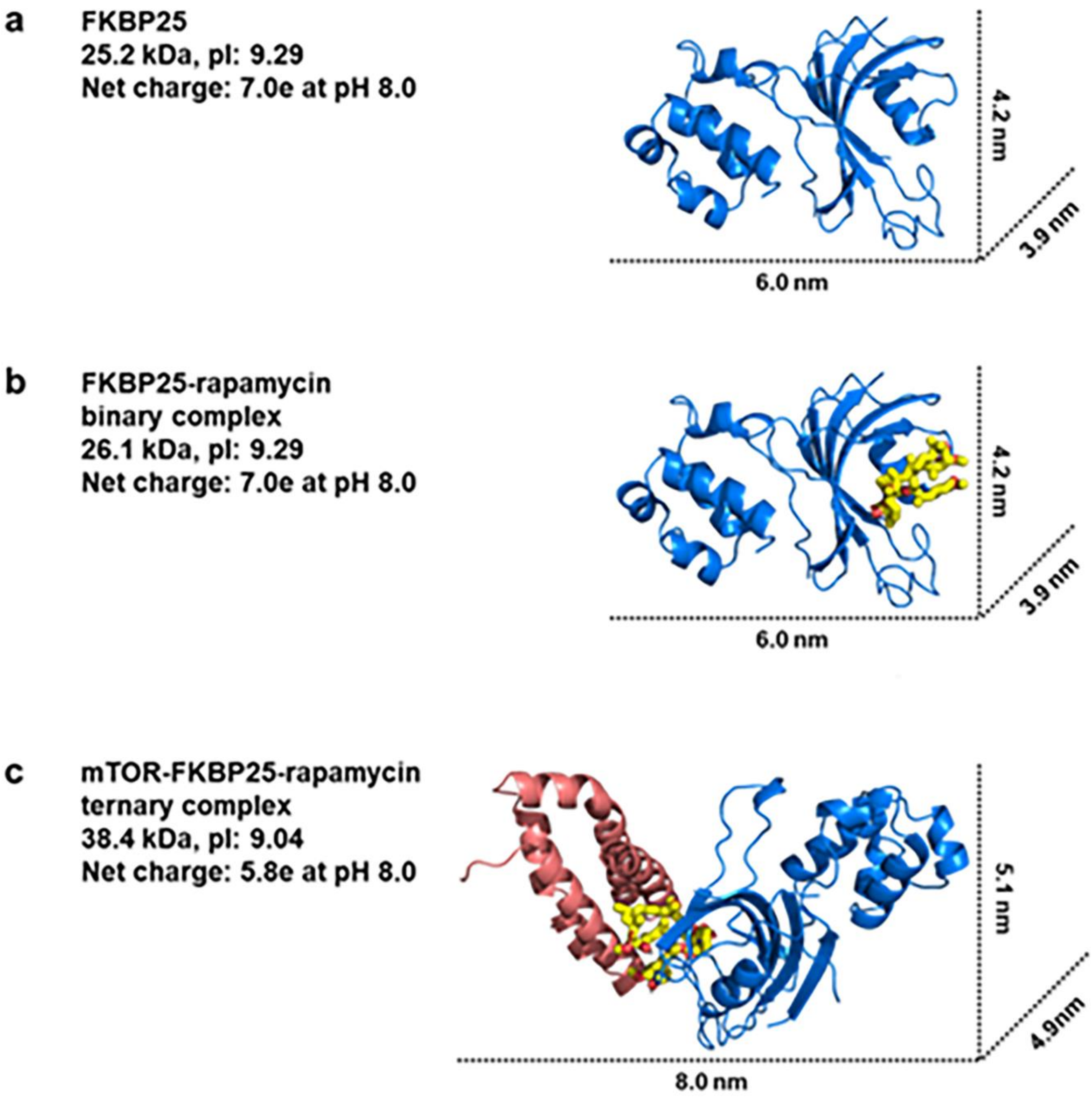

Figure S7. Properties and size dimensions of FKBP25, FKBP25-rapamycin binary complex, and mTOR-FKBP25-rapamycin ternary complex. (a) FKBP25, (b) FKBP25-rapamycin binary complex, and (c) mTOR-FKBP25-rapamycin ternary complex. The dimensions of the proteins were measured by executing the Draw_Protein_Dimensions.py script in Pymol.

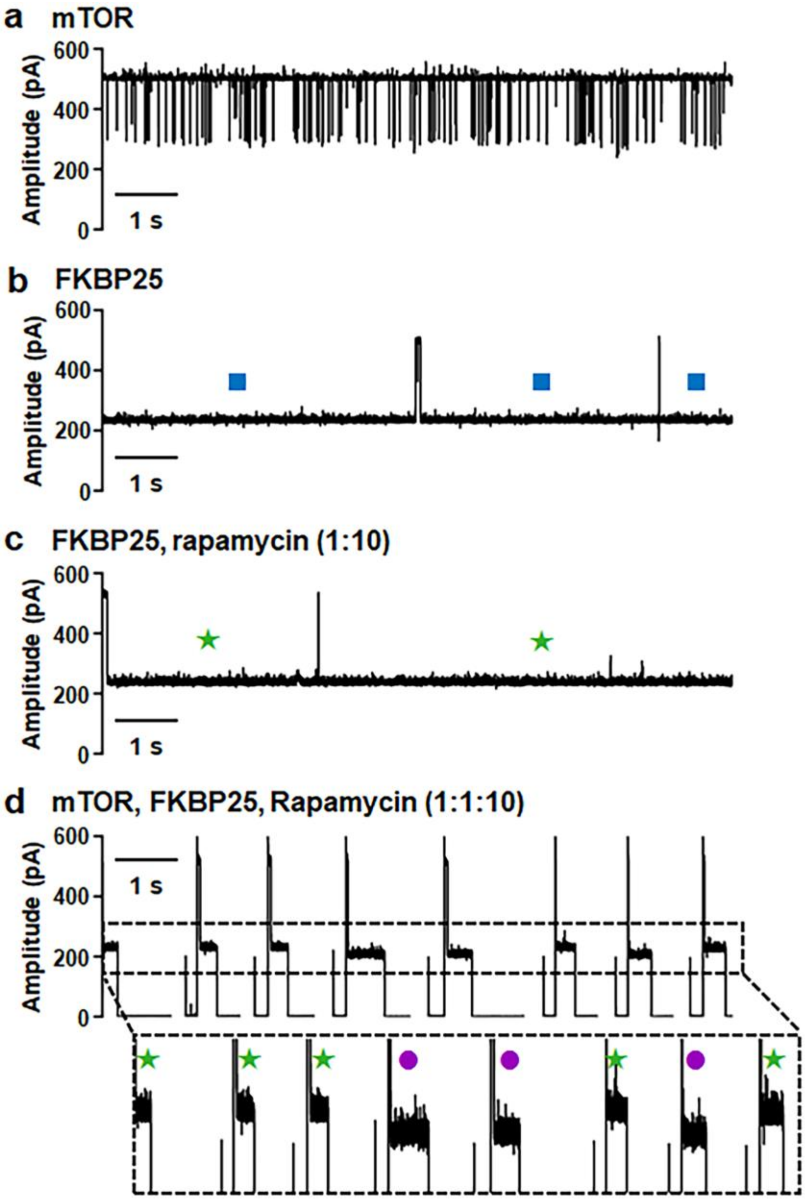

Figure S8. Measurements of current signals from mTOR, FKBP25, FKBP25-rapamycin binary complex, and mTOR-FKBP25-rapamycin ternary complex. (a-d) Representative current traces from the analytes. (a) mTOR, (b) FKBP25, (c) FKBP25-rapamycin (1:10) binary complex, and (d) mTOR-FKBP25-rapamycin (1:1:10) ternary complex. (d) The captured proteins were manually released by zero voltage. Two types of current traces for the binary and ternary complexes (green star and purple circle, respectively) were observed with different current block levels. All the nanopore measurements were conducted at an applied voltage of +60 mV.

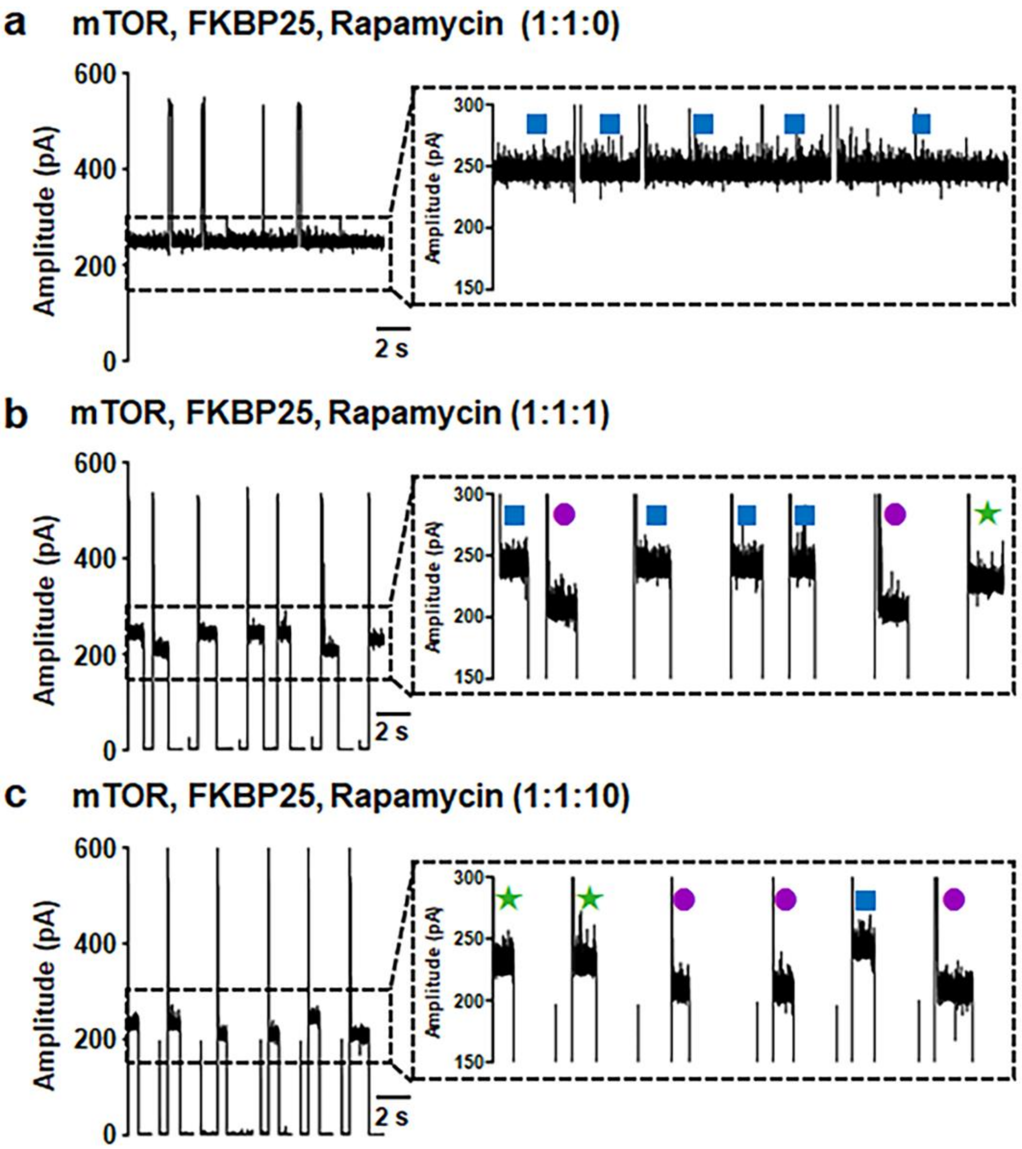

Figure S9. Formation of rapamycin-dependent mTOR-FKBP25-rapamycin ternary complex. (a-c) Representative current traces corresponding to the detection of mTOR and FKBP25 (1:1) in the absence (a) and presence of rapamycin at a 1:1:1 (b) and (c) 1:1:10 ratios. . In the intervals of these events, the captured proteins were manually released by zero voltage. FKBP25 and the binary and the ternary complexes are indicated by blue squares, green stars, and purple circles, respectively. All the nanopore measurements were conducted at an applied voltage of +60 mV.

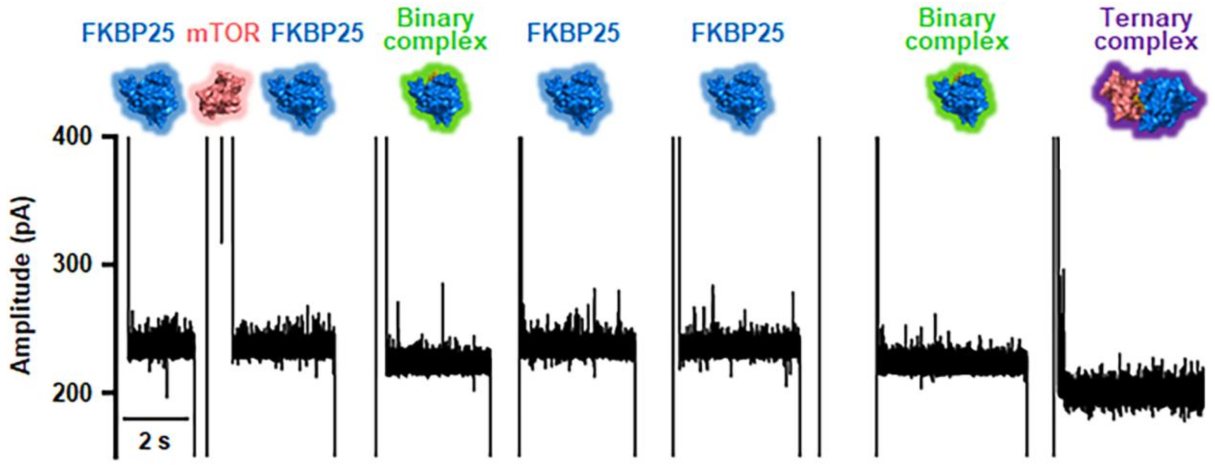

Figure S10. YaxAB nanopore measurements of single proteins and binary and ternary complexes at a low protein concentration of analytes. Representative current trace corresponding to the detection of mTOR, FKBP25, FKBP25-rapamycin binary complex, and mTOR-FKBP25-rapamycin ternary complex. mTOR, FKBP25, and rapamycin were simultaneously treated to the *cis* side of YaxAB nanopores at a concentration of 10 nM (molar ratio of 1:1:1). The captured proteins were manually released by zero voltage. All the nanopore measurements were conducted at an applied voltage of +60 mV.

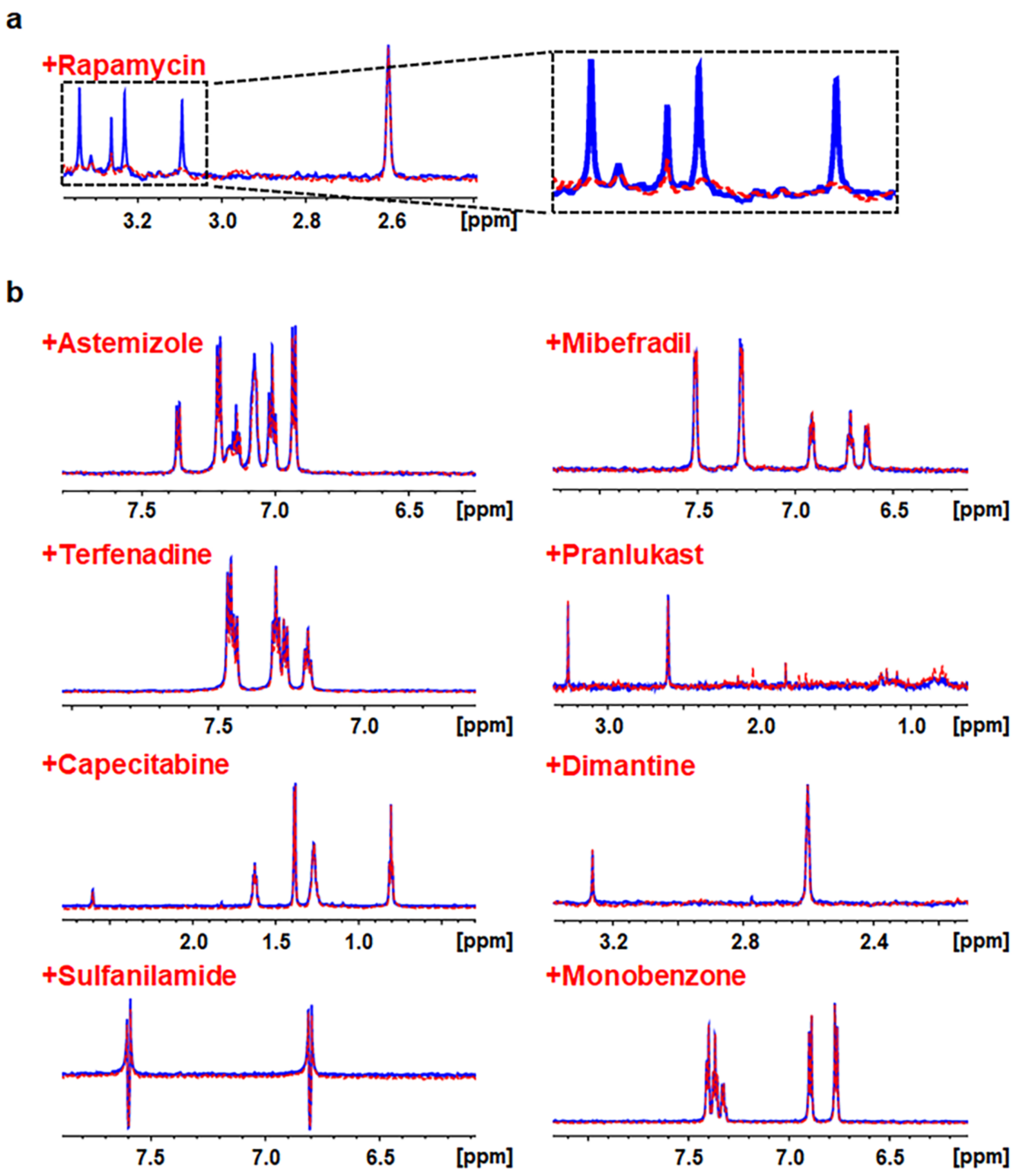

Figure S11. 1D CPMG NMR spectra for multiple compounds. 1D CPMG NMR spectra of rapamycin (a) or non-binding compounds (b) in the absence and presence of mTOR. The blue solid and red dashed lines indicate 1D CPMG spectra of the small molecule compounds in the absence and presence of mTOR protein, respectively.

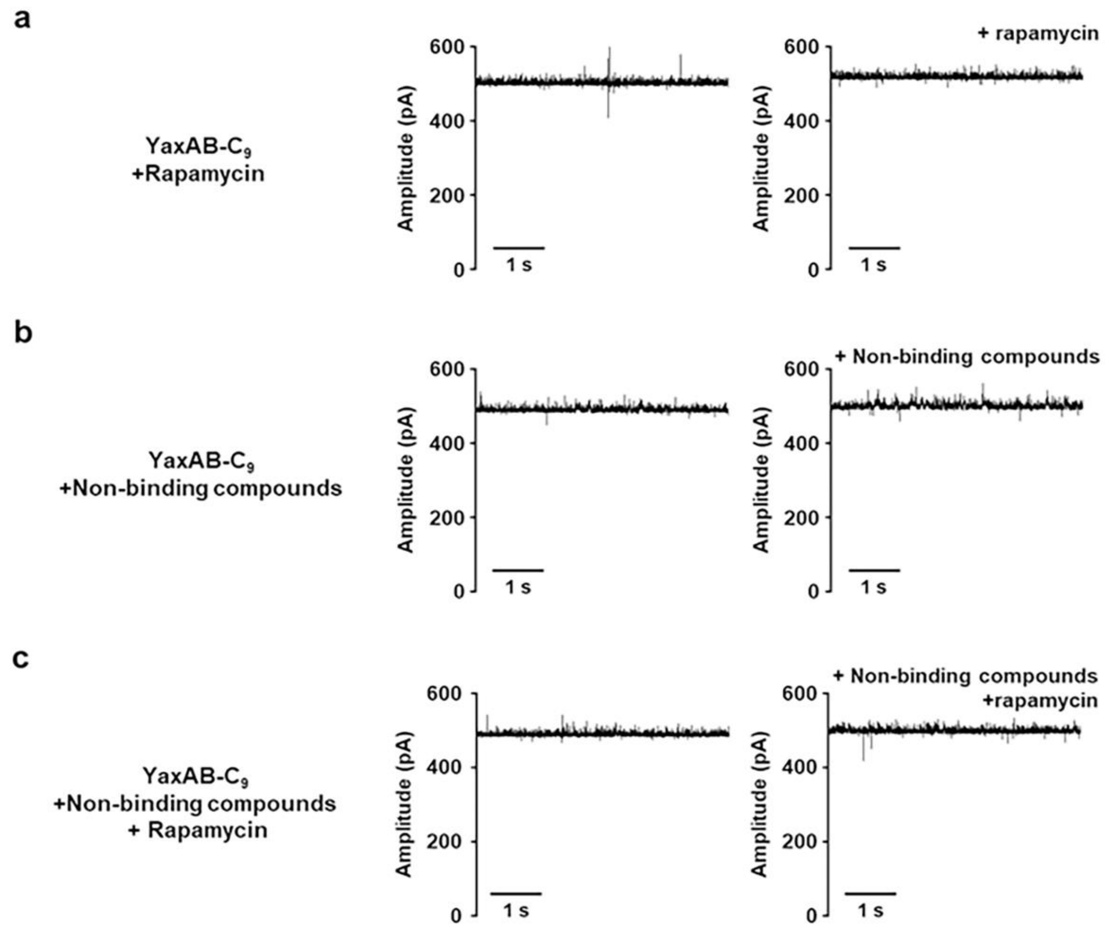

Figure S12. YaxAB nanopore measurements with rapamycin or a mixture of small molecule compounds. (a-c) Representative current traces corresponding to the detection of YaxAB-C_9 nanopores in the absence or presence of rapamycin (a), non-binding compounds (astemizole, terfenadine, capecitabine, sulfanilamide, mibefradil, pranlukast, dimantine, and monobenzone) (b), and a mixture of non-binding compounds with rapamycin (c). All drug compounds were treated at a concentration of 1.6 μM to the *cis* side of YaxAB-C_9 nanopores. All the nanopore measurements were conducted at an applied voltage of +60 mV.

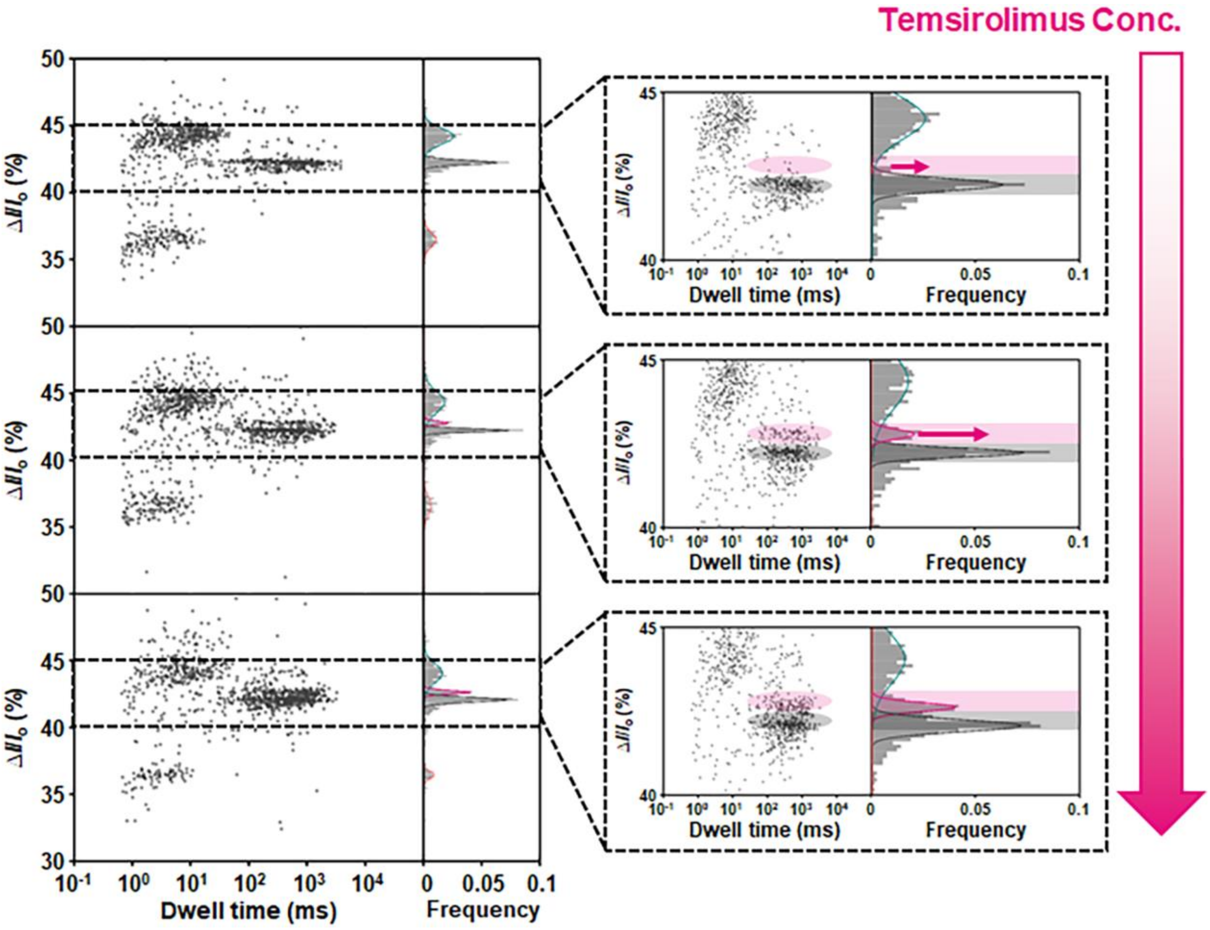

Figure S13. Discrimination between rapamycin- and temsirolimus-induced ternary complexes. Statistical analysis of 2D-scatter plots ($\Delta I/I_o$ versus dwell time) and histograms ($\Delta I/I_o$) corresponding to the detection of mTOR, FKBP12, and rapamycin (1:1:0.5) as a function of increasing concentrations of temsirolimus at 1:1:0.5:0, 1:1:0.5:0.2, and 1:1:0.5:0.5 ratios.
